# Aryl Coenzyme A Ligases, a Subfamily of the Adenylate-Forming Enzyme Superfamily

**DOI:** 10.1128/AEM.00690-21

**Published:** 2021-08-26

**Authors:** M. E. Arnold, I. Kaplieva-Dudek, I. Heker, R. U. Meckenstock

**Affiliations:** a Environmental Microbiology and Biotechnology (EMB), University of Duisburg-Essen, Essen, Germany; Chinese Academy of Sciences

**Keywords:** 2-naphthoate-CoA ligase, 2-phenanthrene-CoA ligase, ANL superfamily, BTEX degradation, PAH degradation, adenylate-forming enzymes, aryl-CoA ligases, benzoate-CoA ligase, hydrocarbon degradation, phenylacetate-CoA ligase

## Abstract

Aryl coenzyme A (CoA) ligases belong to class I of the adenylate-forming enzyme superfamily (ANL superfamily). They catalyze the formation of thioester bonds between aromatic compounds and CoA and occur in nearly all forms of life. These ligases are involved in various metabolic pathways degrading benzene, toluene, ethylbenzene, and xylene (BTEX) or polycyclic aromatic hydrocarbons (PAHs). They are often necessary to produce the central intermediate benzoyl-CoA that occurs in various anaerobic pathways. The substrate specificity is very diverse between enzymes within the same class, while the dependency on Mg^2+^, ATP, and CoA as well as oxygen insensitivity are characteristics shared by the whole enzyme class. Some organisms employ the same aryl-CoA ligase when growing aerobically and anaerobically, while others induce different enzymes depending on the environmental conditions. Aryl-CoA ligases can be divided into two major groups, benzoate:CoA ligase-like enzymes and phenylacetate:CoA ligase-like enzymes. They are widely distributed between the phylogenetic clades of the ANL superfamily and show closer relationships within the subfamilies than to other aryl-CoA ligases. This, together with residual CoA ligase activity in various other enzymes of the ANL superfamily, leads to the conclusion that CoA ligases might be the ancestral proteins from which all other ANL superfamily enzymes developed.

## INTRODUCTION

Thioester, amide, and ester bonds are very common in nature and are often part of typical chemical building blocks. Enzymes catalyzing the formation of these bonds occur in nearly all forms of life. Many of these enzymes belong to the superfamily of adenylate-forming enzymes, which were structured into a novel order of classes and subclasses by S. Schmelz and J. H. Naismith in 2009 (1). Class I of this superfamily is composed of three subclasses: subclass Ia comprises nonribosomal peptide synthetase (NRPS) adenylation domains, subclass Ib includes acyl and aryl coenzyme A (CoA) synthetases/ligases, and subclass Ic includes oxidoreductases (1).

Class I enzymes can serve various functions. For example, they are involved in fatty acid metabolism and transport (2, 3), cell signaling (4), biofilm formation (e.g., in Candida albicans) (5), the synthesis of antibiotic compounds (e.g., in Streptomyces coelicolor) (6), protein transport (2, 7, 8), and many others.

**FIG 1:**
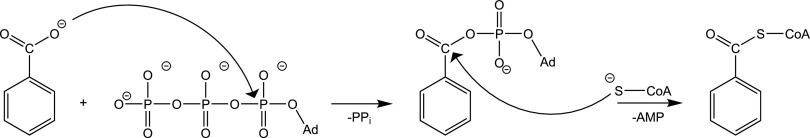
Mechanism of benzoyl-CoA formation by benzoate-CoA ligase. In the first reaction, adenylation, the carboxylic acid of benzoate attacks the α-phosphate of ATP to form a reactive benzoate-AMP intermediate and PP_i_. In the second reaction, thioesterification, the benzoate-AMP intermediate reacts with the thiol group of the CoA and releases AMP by forming the CoA thioester. Ad, adenosyl group.

This review focuses on aryl-CoA ligases (ACLs). ACLs catalyze the formation of a thioester bond between aromatic compounds and CoA using ATP. Their general mechanism is equivalent to that of acyl-CoA ligases, and the first reaction is shared among most adenylate-forming enzymes. In this step, the negatively charged oxygen of the carboxylate substrate performs a nucleophilic attack on the more positive α-phosphorous to form an aryl-adenylate intermediate and release pyrophosphate (PP_i_) (Fig. 1) (for a comprehensive and detailed review of the mechanism, see reference 9; for a review about adenylate-forming enzymes of all classes, see reference 1).

In the second reaction, the intermediate is attacked by a nucleophile, in the case of the aryl-CoA ligases the thiol group of CoA, with AMP as the leaving group (Fig. 1) (1, 10, 11). For 4-chlorobenzoate:CoA ligase, it is proposed that AMP becomes activated as a leaving group by the interaction of its oxygen atoms with hydroxyl groups of two surrounding threonine residues of the enzyme (11).

CoA thioesters are formed by many organisms and serve various purposes. In many bacteria degrading aromatic or polycyclic aromatic compounds, such as, for example, Rhodopseudomonas palustris, the formation of a CoA thioester helps to accumulate substrates inside the cell (12). This accumulation comes to pass for two reasons. First, aromatic substrates entering the cell by diffusion through the membrane are converted to CoA thioesters, maintaining a downhill concentration gradient between the cytoplasm and the outside (12). Second, the CoA group prevents back-diffusion out of the cell due to its bulky size and polar structure (13).

Furthermore, the formation of the CoA thioester activates the substrate for further reactions or degradation steps because the CoA thioester group potentially draws electrons from the aromatic ring, facilitating, for example, the reduction of the ring system (14–16).

In some enterobacteria as well as eucaryotes, membrane-bound acyl-CoA ligases mediate long-chain fatty acid transport into the cell as well as activating the substrate by CoA ligation (3, 17, 18; for a comprehensive review on the role of class I adenylate-forming enzymes in the *trans*-membrane movement of long-chain fatty acids, see reference 2).

## ARYL-CoA LIGASES FROM ANAEROBICALLY AND AEROBICALLY GROWN BACTERIA

Aryl-CoA ligases have been found in a variety of bacteria that grow not only anaerobically but also aerobically with aromatic substrates. All these CoA ligases catalyze a comparable thioesterification reaction that depends on Mg^2+^, ATP, and free CoA. All aryl-CoA ligases are oxygen insensitive and have an alkaline pH optimum of between 7.0 and 9.3 (Table 1).

**TABLE 1:** Characteristics of different aryl-CoA ligases

Aryl-CoA ligase	Microorganism	Growth condition	Native protein form	Subunit size (kDa)^*a*^	pH optimum	*K_m_* (μM)^*b*^	Substrate affinity	Reference
Benzoate:CoA ligase	Rhodopseudomonas palustris	Anaerobic	Monomer	60	8.4–8.9	0.6–2	Benzoate and 2- and 4-fluorobenzoate	44

	Azoarcus evansii	Anaerobic Aerobic	Homodimer Homodimer	120 56 (130)	9.3 9	11 11	Benzoate, 2- ,3-, and 4-fluorobenzoate, and 2-aminobenzoate	25, 26
	Thauera aromatica	Aerobic/anaerobic	Monomer	58	8.5	16	Benzoate, 2- ,3-, and 4-fluorobenzoate, and 2-aminobenzoate	34
	*Magnetospirillum* sp. strain TS-6	Aerobic/anaerobic	Homodimer	60 (120)	9	30	Benzoate and 2- and 4-fluorobenzoate	35


4-Chlorobenzoate:CoA ligase	Pseudomonas sp. strain CBS3	Aerobic	Homodimer	57	7.5	8.5 ± 0.09	4-Chlorobenzoate, benzoate, 4-bromobenzoate, 4-iodobenzoate, and 4-methylbenzoate	45




2-Aminobenzoate:CoA ligase	Azoarcus evansii	Aerobic	Monomer	65	8.5	10	2-Aminobenzoate and benzoate	29

		Anaerobic	Monomer	60	8.5–9.2	13	2-, 3-, and 4-fluorobenzoate	25

4-Hydroxybenzoate:CoA ligase	Rhodopseudomonas palustris	Anaerobic	Dimer	61 (117)	>9	22	4-Hydroxybenzoate, benzoate, and cyclohex-1,4-dienecarboxylate	79


	Thauera aromatica	Aerobic	Monomer	48	8.5	37	4-Hydroxybenzoate and 4-aminobenzoate	36


Phenylacetate:CoA ligase	Pseudomonas putida	Aerobic	Monomer	48 ± 1	7.0–8.5	16.5	Phenylacetate	39
	Azoarcus evansii	Anaerobic	Monomer	52 ± 2	8.5	60		31
		Aerobic	Monomer	52	8–8.5	14		32
	Burkholderia cenocepacia Paak1/Paak2	Aerobic	Homodimer	48 (82)	7.5	62 ± 4/150 ± 7		72

3-Hydroxybenzoate:CoA ligase	Thauera aromatica	Anaerobic	Monomer	60	≥9	60 ± 5	3- and 4-hydroxybenzoate	37

a Subunit sizes in parentheses denote the size of the full dimer.

b *K_m_* values given here refer to the main substrate.

**FIG 2:**
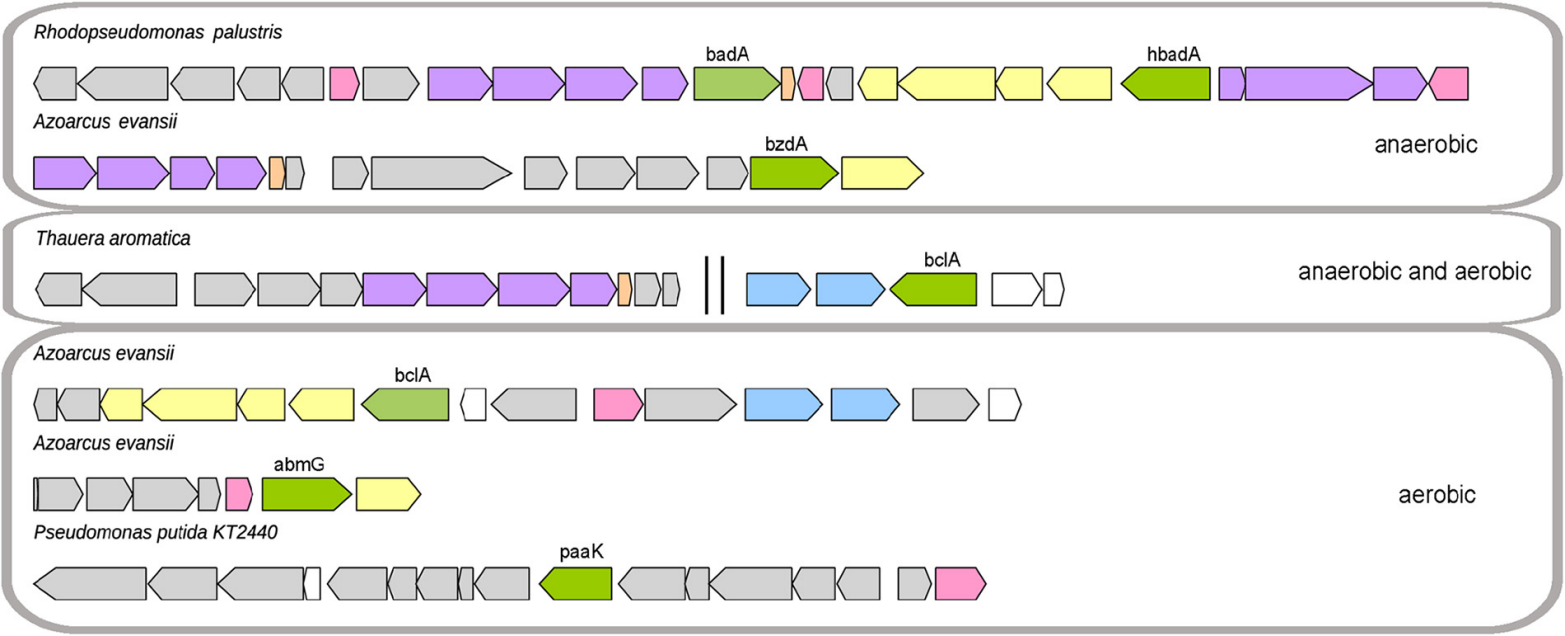
Organization of gene clusters involved in the anaerobic or/and aerobic catabolism of benzoate, 4-hydroxybenzoate, 2-aminobenzoate, or phenylacetate in Rhodopseudomonas palustris, Azoarcus evansii, Thauera aromatica, or Pseudomonas putida KT2440, created using Gene Graphics (80). Genes are represented by tags: green, genes encoding CoA ligases (*badA*, *bzdA*, and *bclA*, benzoate:CoA ligase; *hbadA*, 4-hydroxybenzoate:CoA ligase; *abmG*, 2-aminobenzoate:CoA ligase; *paaK*, phenylacetate:CoA ligase); violet, genes encoding the subunits of the corresponding reductases for anaerobic degradation; orange, genes encoding ferredoxin; pink, regulatory genes; yellow, putative transport genes; gray, genes encoding enzymes for the downstream degradation pathway; white, genes of unknown function. Two vertical lines indicate that the genes are not adjacent in the genome.

Anaerobic degradation of aromatic acids always involves the formation of the corresponding CoA thioester by an aryl-CoA ligase. CoA thioester formation facilitates the uptake of aromatic hydrocarbons (12, 13, 19) and is certainly crucial for further degradation (15, 16, 20, 21). Genes encoding these aryl-CoA ligases are mostly located adjacent to genes for enzymes involved in the anaerobic degradation pathway, such as reductases (Fig. 2) (22).

Bacteria grown aerobically with aromatic substrates usually use oxygenases to activate the aromatic compound through the formation of a hydroxylated intermediate that is oxygenolytically cleaved (23). In several bacteria, however, the aerobic metabolism of aromatic acids occurs via a novel type of aerobic degradation pathway (*box* pathway). These aromatic acids are activated via an aryl-CoA ligase, similar to anaerobic degradation, and are further hydrolytically cleaved (23). It is proposed that the produced CoA thioester acts as an inducer for the expression of the downstream enzymes in aerobic and anaerobic degradation (24).

There are a few bacteria known that are able to degrade certain aromatic acids such as benzoate, 2-aminobenzoate, and also phenylacetate aerobically and anaerobically using aryl-CoA ligases. This is considered an adaptation of these bacteria to changes between anoxic and oxic conditions (16). The denitrifying bacterium Azoarcus evansii is a great example of a bacterium that induces two distinct aryl-CoA ligases for several aromatic acids, depending on the availability of oxygen during growth. Benzoate:CoA ligases expressed in anaerobically or aerobically grown Azoarcus evansii cells are homodimers with different molecular masses (25, 26) and gene locations on the chromosome. One benzoate:CoA ligase, BzdA, is encoded in the cluster coding for enzymes involved in anaerobic degradation (*bzd* cluster), whereas the other one, BclA, is located within the cluster for enzymes required for aerobic degradation (*box* cluster) (Fig. 2) (27, 28). Two other monomeric aryl-CoA ligases were found in A. evansii growing with 2-aminobenzoate: one is induced under oxic conditions (29), and the other is found in anaerobically grown cultures (25). Two very similar clusters encoding enzymes for the aerobic degradation of 2-aminobenzoate, including the 2-aminobenzoate:CoA ligase, are present in the genome (30), while genes involved in anaerobic degradation are barely known. *A. evansii* grown anaerobically on 2-aminobenzoate also expresses a small amount of the aerobically induced 2-aminobenzoate:CoA ligase (25). The expression of both 2-aminobenzoate:CoA ligases during anaerobic growth indicates that *A. evansii* is able to switch rapidly from anaerobic to aerobic degradation of 2-aminobenzoate. Furthermore, this implies that the expression of both ligases is not exclusively under the control of the availability of oxygen and is thus regulated by other factors.

*A. evansii* also expresses two distinct aryl-CoA ligases in aerobic or anaerobic cultures grown with phenylacetate. Both phenylacetate:CoA ligases are monomers, have similar molecular masses and nearly the same pH optima, and are active only with phenylacetate. Furthermore, both ligases require glycerol for stability. Despite this similarity, both phenylacetate:CoA ligases have totally different N-terminal amino acid sequences and are encoded in the vicinity of enzymes required for aerobic or anaerobic degradation pathways, respectively (22, 31–33).

Based on the position of these aryl-CoA ligase genes in the genome of *A. evansii*, it might be that all these aryl-CoA ligases are expressed together with the enzymes required for the corresponding degradation pathway, as has been shown for the aerobically induced 2-aminobenzoate:CoA ligase (30).

In contrast to *A. evansii*, the denitrifying bacteria Thauera aromatica and *Magnetospirillum* sp. strain TS-6 expressed the same benzoate:CoA ligase (BclA) when the cultures were grown aerobically or anaerobically with benzoate (34, 35). The gene for benzoate:CoA ligase of Thauera aromatica was found as a part of the gene cluster for the novel aerobic benzoate oxidation pathway (*box* pathway) and not in the gene cluster for anaerobic benzoyl-CoA metabolism (Fig. 2) (34). In contrast, the *bclA* gene of *Magnetospirillum* sp. is located in neither the *bzd* cluster nor the *box* cluster (35). This indicates that the benzoate:CoA ligase is expressed independently from enzymes involved in the corresponding degradation pathway. Thus, the presence or absence of oxygen induces the expression of the respective downstream enzymes (34, 35). This opportunity may enable these bacteria to survive in environments where the oxygen conditions are changing rapidly and they need to switch between anaerobic and aerobic degradation pathways.

## SUBSTRATE SPECIFICITY

Even though all aryl-CoA ligases utilize the same reaction mechanism, substrate specificities differ widely (Table 1). Eight different aryl-CoA ligases have been found in the denitrifying bacterium *A. evansii* alone: two benzoate:CoA ligases, two 2-aminobenzoate:CoA ligases, two phenylacetate:CoA ligases, a 3-hydroxybenzoate:CoA ligase, and a 4-hydroxybenzoate:CoA ligase (25, 26, 29, 31, 32). Each of these aryl-CoA ligases is encoded in the vicinity of its corresponding downstream genes. *A. evansii* grown anaerobically in a batch culture with 2-aminobenzoate showed the presence of 2-aminobenzoate:CoA ligase, benzoate:CoA ligase, and a small amount of the 2-aminobenzoate:CoA ligase usually produced during aerobic growth. All three aryl-CoA ligases prefer the same substrates (25). Even benzoate:CoA ligase induced under oxic conditions with benzoate as the substrate showed similar substrate affinities (26). This suggests a regulatory function of these ligases in the downstream degradation pathway and, due to the similar substrate specificities, may also allow switching easily from one degradation pathway to another. Interestingly, *A. evansii* expresses four enzymes with nearly the same substrate specificity and not only one as in the denitrifying bacterium T. aromatica. The benzoate:CoA ligase induced during aerobic and anaerobic growth on benzoate or 2-aminobenzoate in T. aromatica has a broad substrate specificity (34). This may enable T. aromatica to alternate rapidly between different degradation pathways depending on the occurrence of the substrates. Two further aryl-CoA ligases were identified in T. aromatica, the distinct monomeric aryl-CoA ligases for the isomers 3-hydroxybenzoate and 4-hydroxybenzoate (36, 37). These two aryl-CoA ligases have a very narrow substrate specificity compared to the benzoate:CoA ligase. This suggests that 3-hydroxybenzoate:CoA ligase and 4-hydroxybenzoate:CoA ligase, in contrast to the benzoate:CoA ligase, are involved in the regulation of the downstream genes and are able to activate only their corresponding degradation pathway.

The phototrophic bacterium R. palustris grown with 4-hydroxybenzoate in the absence of oxygen showed 4-hydroxybenzoate:CoA ligase activity. In contrast to the high substrate specificity of the 4-hydroxybenzoate:CoA ligase of T. aromatica, this ligase has a broad substrate affinity. The gene for 4-hydroxybenzoate:CoA ligase (*hbadA*) is located adjacent to the gene clusters coding for enzymes involved in 4-hydroxybenzoate degradation as well as enzymes required for benzoate degradation (Fig. 2) (38). This high substrate diversity may enable R. palustris to also regulate the expression of enzymes required for the downstream degradation of other aromatic acids such as benzoate.

A highly substrate-specific aryl-CoA ligase is the phenylacetate:CoA ligase induced in *A. evansii* and Pseudomonas putida when grown with phenylacetate. The aerobically and anaerobically induced phenylacetate:CoA ligases from *A. evansii* and the aerobically induced one from P. putida could convert only phenylacetate to phenylacetyl-CoA. No other substrates were converted to their corresponding CoA thioesters (31, 32, 39), indicating a potential regulatory function in the expression of enzymes exclusively involved in the downstream degradation of phenylacetate.

This shows that the substrate specificity of monoaromatic aryl-CoA ligases differs from ligase to ligase, even if they are expressed during growth with the same substrate; this may indicate different regulatory functions.

The substrate specificity of polycyclic aromatic aryl-CoA ligases is under investigation. The polycyclic aromatic aryl-CoA ligases expressed in the naphthalene-degrading, sulfate-reducing culture N47 (40) or NaphS2 (41) showed broad substrate specificity. They can convert fluorinated naphthoates to their corresponding CoA thioester at the same rate as naphthoate, similar to what is known for all benzoate:CoA ligases. Several hydroxylated naphthoates were also used by the naphthoate:CoA ligase but with lower conversion rates (M. E. Arnold, F. Kaschani, and R. U. Meckenstock, unpublished results). In the sulfate-reducing, phenanthrene-degrading enrichment culture TRIP1 (42, 43), the phenanthroate:CoA ligase is very specific for 2-phenanthroate. Only 3-phenanthroate was utilized by the phenanthroate:CoA ligase as well but with a much lower conversion rate (I. Kaplieva-Dudek, F. Kaschani, and R. U. Meckenstock, unpublished results).

## INFLUENCE OF CATIONS AND THIOL GROUP-MODIFYING AGENTS ON ARYL-CoA LIGASE ACTIVITY

All known adenylate-forming enzymes require magnesium ions for their catalytic reaction. The absence or replacement of magnesium by other cations leads to a loss of enzyme activity. For example, the addition of the known inhibitor of magnesium-dependent enzymes NaF to phenylacetate:CoA ligase of *A. evansii* decreased the ligase activity by up to 20% (31). The 4-chlorobenzoate:CoA ligase of Pseudomonas sp. strain CBS3 has 12-fold-lower activity without Mg^2+^ (11), indicating that magnesium is necessary for optimal enzyme activity. Magnesium ions neutralize the charge of ATP and later the charge of the leaving group pyrophosphate. Furthermore, magnesium ions can interact with and stabilize the acyl-AMP intermediate. In nearly all aryl-CoA ligases, Mg^2+^ can be replaced only by Mn^2+^ without loss of activity (25, 29, 31, 39, 44). For the 4-chlorobenzoate:CoA ligase from Pseudomonas sp. strain CBS-31, it was also shown that Mg^2+^ can be replaced by Co^2+^ as a cofactor (45). In most cases, other cations such as Zn^2+^, Cu^2+^, Ni^2+^, Hg^2+^, and Mo^5+^ have strong inhibitory effects on ligase activity. Copper ions can probably interact with the thiol group and thus prevent the CoA from binding to the substrate. Monovalent cations like K^+^, Na^+^, Li^+^, and Rb^+^ have no effect on aryl-CoA ligase activity (25, 29, 31, 39, 44). In contrast to monocyclic aromatic aryl-CoA ligases, polycyclic aromatic aryl-CoA ligases were also active with either Na^+^ or K^+^ instead of Mg^2+^ when using cell extracts (Arnold et al., unpublished; Kaplieva-Dudek et al., unpublished). However, it cannot be excluded that a sufficient amount of Mg^2+^ is present in the ligase assays since a cell extract was used and not purified enzymes. So far, similar findings have also been reported for xenobiotic/medium-chain fatty acid:CoA ligases of bovine liver mitochondria and cinnamate:CoA ligase of Hypericum calycinum cell cultures (46–48). The phenanthroate:CoA ligase from the sulfate-reducing, phenanthrene-degrading culture TRIP1 showed higher ligase activity when Na^+^ or K^+^ was added to cell extracts instead of Mg^2+^. With a combination of Mg^2+^ and K^+^, the greatest conversion of 2-phenanthroate was measured. Furthermore, phenanthroate:CoA ligase was inhibited with increasing Mg^2+^ concentrations (Kaplieva-Dudek et al., unpublished). This indicates a difference between aryl-CoA ligases converting monocyclic aromatic and polycyclic aromatic hydrocarbon (PAH)-carboxylic acids regarding the influence of cations on ligase activity. Other strong inhibitory effects on monocyclic aromatic aryl-CoA ligases were detected by thiol group-modifying agents such as *N*-ethylmaleimide, iodoacetamide, *p*-hydroxymercuribenzoate, 5,5′-dithiobis-(2-nitrobenzoic acid), and *p*-chloromercuribenzoic acid, which indicates that a free thiol group of the CoA is necessary for enzyme activity (25, 32, 36).

## ARYL-CoA LIGASES FORM TWO MONOPHYLETIC CLADES

A neighbor-joining phylogenetic tree of amino acid sequences of the adenylate-forming enzyme superfamily (ANL superfamily) was recently constructed by L. Clark et al. (49), aligning in total 374 protein sequences of the ANL superfamily, of which 49 belonged to aryl-CoA ligases. The unrooted tree classified the proteins into nine distinct groups: luciferases, three groups of fatty acyl-CoA synthetases, nonribosomal peptide synthetases (NRPSs), fatty acid-AMP ligases, methylmalonyl-CoA synthetases, mycobacterial FadD10 long-chain fatty acyl-CoA ligases, and aryl-CoA ligases. The alignment of the sequences yielded 5 amino acids conserved in all groups: Glu328, Gly384, Asp418, Arg433, and Lys524 (residue positions correspond to the Thermus thermophilus sequence as noted in this paper). Conserved residues were mostly found surrounding the active site of the enzymes containing the AMP- and CoA-binding site rather than the protein surface. Group-specific residues were identified using the GEnt program (50), calculating the conservation of specific residues inside the aryl-CoA group of proteins versus the whole alignment. Two of the eight identified residues interacted directly with the substrate: Asn411 of the 4-chlorobenzoate:CoA ligase from *Alcaligenes* forms a hydrogen bond to the α-phosphate of AMP in the thioesterification conformation, and His207 interacts with the substrate during the adenylation reaction.

Recently, Robinson et al. (51) used a machine-learning approach to identify enzymes of the ANL superfamily and construct a maximum likelihood phylogenetic tree of the characterized proteins. This tree shows that β-lactone synthetases, NRPSs, as well as luciferases form very distinct monophyletic clades. In contrast to the study by L. Clark et al. (49), especially the aryl/acyl-CoA ligases are distributed widely throughout the phylogenetic clades and show a closer relationship to the enzyme subfamilies than to each other. Hence, aryl/acyl-CoA ligases seem to be clearly polyphyletic, and those authors conclude that the ANL superfamily developed from enzymes with an active site comparable to that of modern aryl/acyl-CoA ligases using CoA-SH as a cofactor. In this case, divergent evolution from ancestral CoA ligase-like scaffolds led to the diverse specialized functions of the ANL superfamily. A similar radial topology and common ancestor have been shown previously for the nitroreductase superfamily, where evidence suggests divergent evolution from an ancestral minimal flavin-binding scaffold (52). This line of reasoning is supported by experiments showing low-level CoA-ligase activity in members of the ANL superfamily, specialized primarily toward other functions. Five NRPS A domains were bifunctional, showing CoA ligase activity (53), similar to luciferases (54). Moreover, the fact that single point mutations were able to restore CoA ligase activity in ANL superfamily members strengthens the hypothesis of an ancestral CoA ligase-like scaffold (51). Nonetheless, the alternative of CoA ligase activity developing independently at several times throughout ANL superfamily evolution, as shown with other superfamilies, cannot be conclusively excluded at this point.

**FIG 3:**
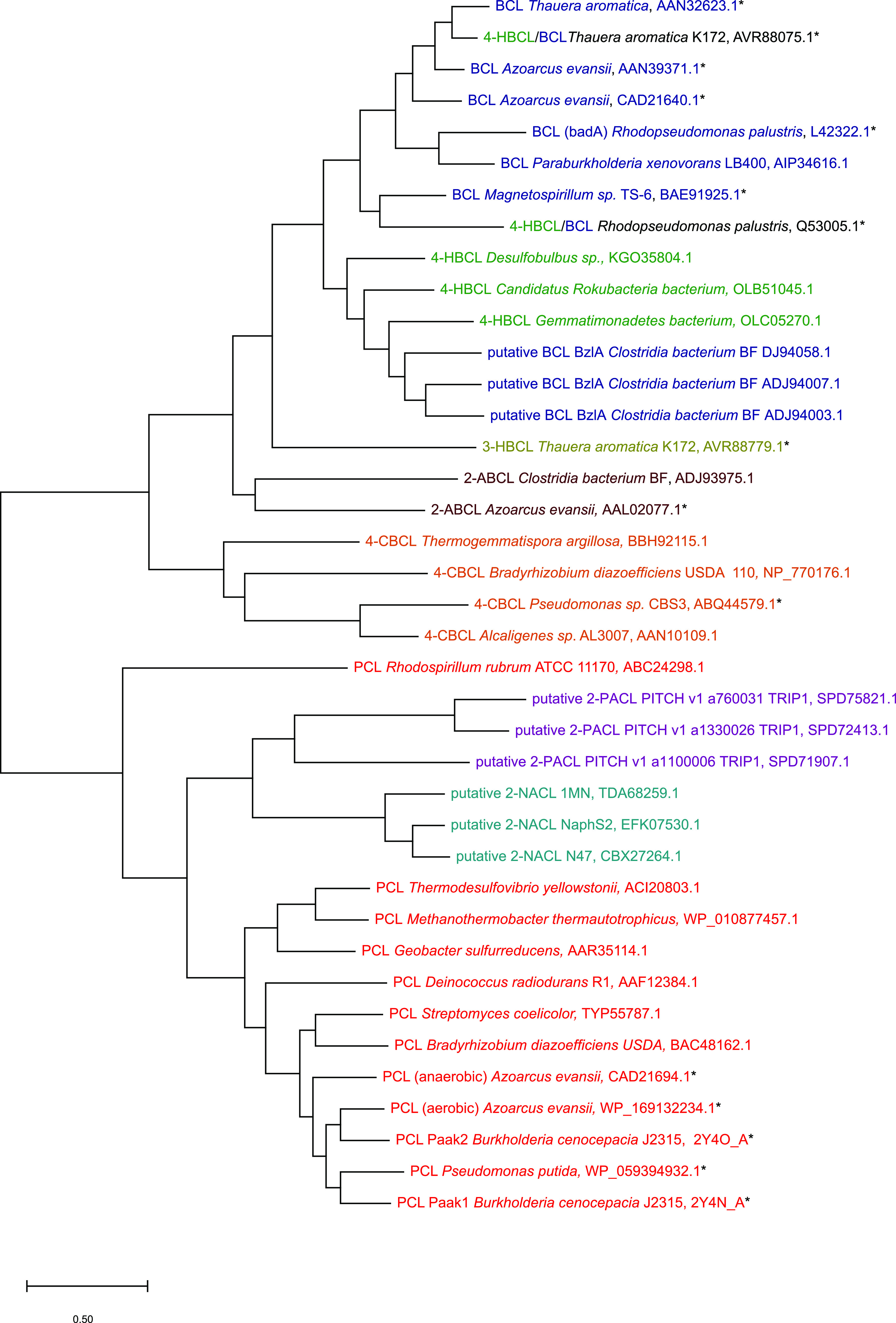
Maximum likelihood phylogenetic tree of aryl-CoA ligases of the ANL superfamily. The tree was constructed in Mega-X using 39 amino acid sequences of the aryl-CoA enzyme family (see GenBank accession numbers at the right). Enzymes marked with an asterisk are listed in Table 1. BCL, benzoate:CoA ligase; 4-HBCL, 4-hydroxybenzoate:CoA ligase; 3/4-CBCL, 3/4-chlorobenzoate:CoA ligase; 2-ABCL, 2-aminobenzoate:CoA ligase; 2-PACL, 2-phenanthroate:CoA ligase; 2-NACL, 2-naphthoate:CoA ligase; PCL, phenylacetate:CoA ligase.

We constructed a maximum likelihood phylogenetic tree with Mega-X (55, 56) using 39 amino acid sequences of the aryl-CoA enzyme family (Fig. 3). Here, the group of the aryl-CoA ligases was divided into two phylogenetically distinct subgroups: benzoate:CoA ligase-like enzymes and phenylacetate:CoA ligase-like enzymes. In this tree, not only do benzoate:CoA ligases and phenylacetate:CoA ligases cluster into separate clades, but putative polycyclic aromatic compound ligases for naphthoate (57, 58) and phenanthroate (43) are also part of the phenylacetate:CoA ligase-like monophyletic clade. The only relatively conserved residues of the aryl-CoA ligases, leading to clustering according to their substrate spectrum inside these two major phylogenetic clades, can be found surrounding the substrate-binding pocket (49).

## STRUCTURAL FEATURES AND CONSERVED RESIDUES OF ARYL-CoA LIGASES

Overall, benzoate:CoA ligase-like and phenylacetate:CoA ligase-like enzymes conform to the general two-domain structure of ANL enzymes, with a larger N-terminal domain with parallel β-sheets forming the core surrounded by α-helices and the active site situated in the linker region between the N- and C-terminal domains. A 140° rotation of the C-terminal domain enables the enzyme to catalyze the two distinct reactions of adenylation and thioesterification (9, 59). During the first reaction, the enzyme adopts the adenylation conformation when ATP and the carboxylate bind to the active site in the N-terminal region. This configuration places the carboxylate in the ideal position to attack the α-phosphate, which leads to the release of PP_i_ and the formation of the adenylate intermediate (60). For the second reaction, a rotation of the C-terminal domain is necessary because the pantetheine tunnel, which is part of the CoA-binding site, is blocked in the adenylation conformation. The pantetheine or thiol group of CoA needs to be close to the intermediate to form the thioester bond and displace the AMP. During the C-terminal domain rotation and the subsequent formation of the thiolation conformation, the pantetheine tunnel is open, and CoA can be placed correctly into the binding pocket, leading to the formation of the aryl-CoA product (61).

The study of the adenylation domain of NRPSs yielded 10 conserved motifs, 9 of which can be found in the wider ANL superfamily (A1 to A8 and A10) (9, 62). Three of these motifs have been previously described (63). A recent publication by L. Clark et al. (49) compared 374 amino acid sequences of the ANL family and found 10 conserved motifs, corresponding partially to the previously established motifs. A comparison of these different motifs and their function can be found in Table 2.

**TABLE 2:** Conserved sequence motifs in the ANL superfamily^*a*^

Motif described by Clark et al.^*b*^	Motif described by Marahiel et al.,^*c*^ A1–A10, and Chang et al.,^*d*^ I–III	Amino acid sequence^*e*^		Function(s) and result(s) of mutation^*f*^	Motif found in aryl-CoA ligases^*g*^
		1–10	A1–A10 (I–III)		
	A1		Ψ(S/T)Ωx(E/Q)Ψ	Structural	
6		(R/K)LANAlxxxLG(V/I/L)K(K/P)GD(R/V)V(A/G)(L/V/I)L			
10	A2	(A/I)GA(V/I)VVP(L/I)NPRx_7_(Y/L)TPK(E/D)(I/L)xYR(L/I)N	(R/K/F)ΨGΨ	Structural	
3	A3 (I)	(T/S)SG(T/S)TGLPKGV(M/L)(L/H)(T/S)H	ΨΨx(S/T)(S/T/G) G(S/T)TGxPK	P-loop	X (Gly189)
				Thr161, 2,000-fold-lower activity	
				Gly163, 1,000-fold-lower activity	
				Gly166, 14-fold-lower activity	
				Pro168, no activity	
				Lys169, 4-fold-lower activity	
	A4		Ω	Aromatic residue, part of the acyl-binding pocket in the active center	
				His207, 500-fold-lower activity	
9		(I/L)(E/Q)K(Y/E)(K/R)(V/I)Tx(L/F)xG(V/A)PTIYR(F/A)L(L/A)(K/Q)			X (Pro278)
7		DLSSL(R/K)xLVS(G/A)(G/A)(A/E)(P/A)LN(P/K)E(V/L)xE			X
	A5 (II)		Ω(G/W)x(A/T)E	Positioning of ATP and binding of Mg^2+^	X
				Tyr304, no change in activity	
				Thr307, 100-fold-lower activity	
				Glu306, 50-fold-lower activity	
8		ExKPGSVG(K/R)(P/V)VP(G/N)V(E/D)V(K/R)(I/V/L)(V/I)DP			
5	A6	GE(I/L)C(V/I)(R/K)x_5_GPG(V/I/L)(M/F/A)KGY(W/Y/L)N	GEx_10–14_GY	Structural	
1	A7 (III)	(Y/L/F)H(T/S)GD(L/I)(G/A)(Y/R)xDEDGY(F/L)(W/F)(I/F)(V/T) (G/D)Rx(K/D)D(L/V)I(K/I)S(G/K/S)G(Y/E/F)(R/N/Q)(I/V)GPAE(I/V/L)ESAL	(S/T)GD	Positioning of ATP, binding/interacting with the ribose hydroxyls of ATP	X (Arg424)
	A8		Rx(D/K)x_6_G	Asp385, 500-fold-lower activity	
				Arg400, 100-fold-lower activity	
4		HPA(V/I)A(E/D)AAV(V/I)G(V/I)P(D/H)(P/E)x(W/A/R)G(E/Q)V(P/V)			
2	A10	P(R/D)x(V/I)(E/V)FUDE(L/I)PK(T/N)(P/A)(S/T)GKI(L/D)(R/K) (R/K)ELR	Px_4_GKΨx(R/K)		X (Lys518)

a A1–A10, conserved motifs of the adenylation domain of NRPS (62); I–III, conserved sequences of the acyl ligase family (63); 1–10, conserved motifs of the ANL superfamily (49); P-loop, phosphate-binding loop.

b See reference 49.

c See reference 62.

d See reference 63.

e Ω, aromatic amino acid (F, Y, H, or W); Ψ, aliphatic amino acid (A, V, L, I, or M); x, any amino acid.

f See references 9, 11, and 63.

g Amino acids used in the phylogenetic analysis of aryl-CoA ligases (Fig. 3) were aligned using the MUSCLE algorithm (Mega-X) and examined for conserved sequences. The 100% conserved amino acids and their positions in the benzoate:CoA ligase of Thauera aromatica (GenBank accession number AAN32623.1) are written in three-letter code. A motif was considered present if 50% of the amino acid code was concurrent with the described motifs.

Crystal structures of the 4-chlorobenzoate:CoA ligase elucidated the role of conserved amino acids in these motifs (64, 65). One of the most conserved regions found in all enzymes of this superfamily is the ATP-binding site. Motif A7, containing a conserved aspartic acid residue, as well as motif A8, containing an arginine residue, interact with ribose hydroxyls. The ATP molecule is further stabilized via the A5 motif. In the 4-chlorobenzoate:CoA ligase, an aromatic tyrosine is the key residue for this interaction. The key catalytic residue lysine in motif A10 interacts in the adenylation conformation of the enzyme with the α-phosphate during the nucleophilic attack on ATP. This lysine is highly conserved in all aryl-CoA ligases and throughout the acyl-CoA and fatty acid acyl-CoA ligases. The lysine residue is the target of acetylation as a posttranslational regulation mechanism (66, 67). Reversible lysine acylation in prokaryotes is a relatively new field of study. The earliest mention of this regulatory mechanism in bacteria was in 2002 (68). The benzoate:CoA ligase BadA from Rhodopseudomonas palustris is one example of regulation through the interaction of acetyltransferases and deacetylases (66). Another, possibly related regulatory mechanism was found in *Azoarcus* sp. strain CIB. Here, the genes for benzoate degradation are located in one operon. Gene expression is regulated by the repressor BzdR, which is dependent on the benzoyl-CoA concentration in the cell (69). Posttranslational lysine acetylation might be a possible way to regulate not just ligase activity but also the expression of aryl-CoA ligase genes through the benzoyl-CoA concentration. This might provide a diverse toolset for bacteria to regulate an energy-consuming reaction.

**FIG 4:**
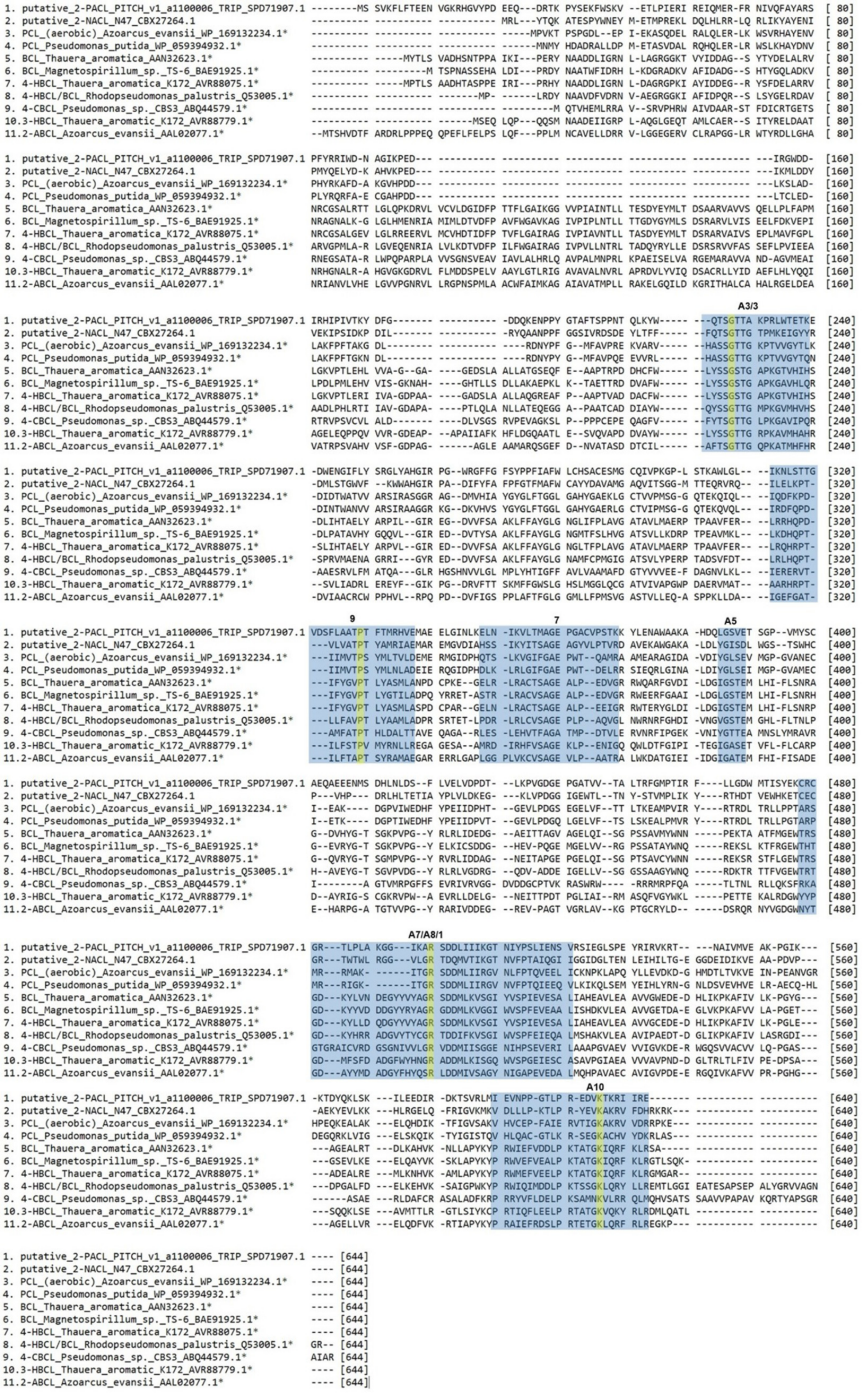
Alignment of 11 out of 39 aryl-CoA ligase protein sequences, with structural motifs highlighted in blue. The protein sequences were taken out of the alignment of 39 aryl-CoA ligases used for the maximum likelihood phylogenetic tree in Fig. 3. The whole alignment can be found in the supplemental material. The structural motifs found in aryl-CoA ligases (Table 2) are highlighted in blue. Amino acids that are 100% conserved in the whole alignment are marked in yellow.

The position of the CoA-binding site is not well conserved within the group of aryl-CoA ligases, but it consists of a nucleotide-binding site on the protein surface and a pantetheine tunnel interacting with motif A8. The substrate-binding site within this group shows almost no conservation and is highly diverse even between enzymes with the same main substrate (9). The alignment created for the phylogenetic tree (Fig. 3) has been searched for the motifs described here (Table 2). The sequence alignments containing at least 50% sequence identity with these motifs are marked in Fig. 4.

**FIG 5:**
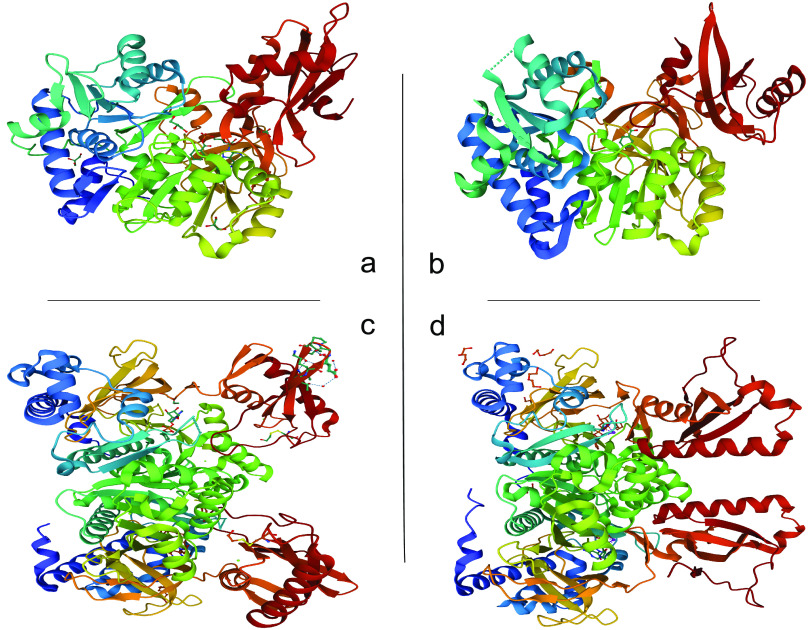
Crystal structures of benzoate:CoA ligases (a and b) and phenylacetate:CoA ligases (c and d). (a) Crystal structure of benzoate:CoA ligase (BadA) from Rhodopseudomonas palustris with benzoyl-AMP (PDB accession number 4ZJZ) (46). (b) Crystal structure of benzoate:CoA ligase from Paraburkholderia xenovorans LB400 (PDB accession number 2V7B) (47). (c and d) Crystal structures of the phenylacetate:CoA ligases PaaK1 (PDB accession number 2Y4N) and PaaK2 (PDB accession number 2Y4O) from Burkholderia cenocepacia J2315 with phenylacetyl adenylate (49). Images were created using Mol* Viewer (78) from the RCSB PDB database.

C. K. Thornburg et al. (59) elucidated the structure of the benzoate:CoA ligase BadA from R. palustris (Fig. 5) and determined the reaction mechanism and wild-type substrate specificity through mutations of the active center. BadA shows a preference for *ortho*-substituted substrates in contrast to *meta* or *para* isomers. Steric effects influence kinetics more than electrostatic interactions. The enzyme shows a two-domain structure with the N-terminal domain containing the benzoate-binding site, typical for the ANL superfamily. The substrate is centered in the active site with the help of charged interactions between a lysine residue contained in the C-terminal domain and the carboxylate group of the benzoate. Contrary to other benzoate:CoA ligases, which adopt the adenylation conformation during cocrystallization with benzoate (65, 70), BadA takes on the thiolation conformation even in the absence of CoA. This indicates two possible subgroups of benzoate:CoA ligases: enzymes with a resting state in the thiolation conformation and those in the adenylation conformation. Those authors propose a multistep reaction mechanism for BadA, analogous to that of the human medium-chain acyl-CoA synthetase ACSM2A (71). Here, the carboxylate binds to the enzyme in the thiolation conformation, followed by a domain rearrangement to the adenylation conformation in which ATP binds to the substrate and acyl-AMP is formed. The last step is the typical domain movement to the thiolation conformation for the thioesterification reaction with CoA.

Crystallization studies of the phenylacetate:CoA ligases PaaK1 and PaaK2 from Burkholderia cenocepacia J2315 (72) (Fig. 5) show the phenylacetate:CoA ligases as a distinct subgroup of the aryl-CoA ligases besides the benzoate:CoA ligases. PaaK1 and PaaK2 show structures similar to those of homologous aryl-CoA ligases apart from one additional feature in the N-terminal domain of these enzymes, a novel microdomain of ∼70 residues. This microdomain is built with a leucine-zipper-like organization and forms a continuous three-helical bundle structure. The microdomain is stabilized by the hydrophobic interactions of 6 leucine residues and Tyr30, Phe44, and Phe63 of PaaK1. This alteration in the N-terminal domain leads to a change in the typical conformation of the group of four β-sheets surrounded by five α-helices as can be found in the benzoate:CoA ligase from Burkholderia xenovorans (70) or the 4-chlorobenzoate:CoA ligase of Pseudomonas sp. (63). The monomeric N-terminal domain of PaaK1 is comprised of three β-sheets surrounded by nine α-helices, which leaves the phosphate-binding loop (P-loop) (motif A3) isolated, leading to the necessary dimerization of this domain. Cocrystallization of PaaK1 with ATP further proved the binding of the phosphates from ATP to the P-loop. Hydrogen bonds formed between the amide backbone of the P-loop and the phosphates from ATP reinforce this coupling, with additional stabilization through hydrogen bonds with Ser94, Thr96, and Thr97. As mentioned above, the lysine residue of the A10 motif is highly conserved in these two enzymes as well as in other aryl-CoA ligases.

## PHENYLACETATE:CoA LIGASES, AN EXPANDING GROUP

Recent studies have elucidated new enzymes, expanding the group of phenylacetate:CoA ligases with substrates outside the already known aryl carboxylates. The 2-hydroxyisobutyric acid:CoA ligase from Aquincola tertiaricarbonis L108 (73) shows a closer relationship to the phenylacetate:CoA ligases PaaK1 and PaaK2, with 34% and 33% coverages of homologous sequences, compared to 20% with other short- and medium-chain acid ANL ligases. The enzyme shows a similar tertiary structure typical of phenylacetate:CoA ligases, forming a dimer with a seven-stranded β-sheet connecting the two subunits. The acyl-binding pocket contains the unique active-site residues Tyr164 and Ser239, leading to a smaller and more polar environment.

A unique set of enzymes from the benzoxazole biosynthesis pathway has recently been identified as a subset of the phenylacetate:CoA ligase family (74, 75). The enzymes AjiA1 and the closely related ligase NatL2 show 30% sequence identity with PaaK1 but do not undergo a domain alteration; instead, these newly described enzymes feature a domain-swapping reaction involving a C-terminal loop constricting the enzymes to one conformation.

As described above in this review, phylogenetic analyses of putative 2-phenanthroate:CoA and 2-naphthoate:CoA ligases (43, 76, 77) (Fig. 3) indicate that they belong to the monophyletic clade of phenylacetate:CoA ligases. These ligases show a sequence similarity of ∼20 to 25% with the described benzoate:CoA ligases, compared to ∼30% sequence similarity with phenylacetate:CoA ligases.

## CONCLUSION

The group of aryl-CoA ligases is a central group of the ANL superfamily. Recent evidence even suggested an aryl-CoA ligase-like enzyme as an ancestral enzyme from which the more specialized functions of the ANL superfamily have evolved (51).

Most aryl-CoA ligases are encoded either in the same operon as or close to the corresponding degradation genes of their respective pathways (22). The regulation of these genes is still not fully understood, as some aryl-CoA ligases are regulated by oxygen availability (24), while others are regulated by posttranslational acetylation of a C-terminal conserved lysine residue (66). This in turn influences benzoyl-CoA levels and might be used in some cases to regulate the gene expression of aryl-CoA ligases through a transcription repressor (69).

Acetylation of catalytic residues in prokaryotes is still a relatively new topic, and as such, further research is necessary. It is still unclear what factors influence the expression of acetyltransferases and deacetylases, as many times, these enzymes are not encoded near their corresponding genes. In the future, more aryl-CoA ligases as well as other ANL superfamily enzymes need to be examined for signs of acetylation, as the lysine residue is highly conserved throughout the family.

Crystallization studies give further insights into and elucidate the key residues responsible for reaction mechanisms while also showing new strategies emerging from newly identified members of this group. The aryl-CoA ligases are divided into two major groups, the benzoate:CoA ligase-like and the phenylacetate:CoA ligase-like enzymes, with their members clustering according to their substrate specificity, as the active site contains most of the conserved amino acid residues (9).

The newly discovered ligases from anaerobic polycyclic aromatic hydrocarbon degradation pathways fall into the second group of phenylacetate:CoA ligases, which expands our knowledge of the essential requirements of these enzymes. In recent years, the group of aryl-CoA ligases has been steadily growing, with more and more enzymes being identified. These enzymes share little sequence similarity, but newly emerging bioinformatic tools and the expansion of machine-learning approaches demonstrate new ways of identifying possible candidate enzymes (51). Future studies will have to focus on these enzymes and their role in the aryl-CoA ligase family as well as the importance of this group in the context of the wider ANL superfamily.
