# Supplementary material for: Aryl Coenzyme A Ligases, a Subfamily of the Adenylate-Forming Enzyme Superfamily
Source: Appl Environ Microbiol. 2021 Aug 26;87(18):e00690-21. doi: 10.1128/AEM.00690-21 (PMC8388817; doi:10.1128/AEM.00690-21)
Supplement: Supplemental file 1 — Supplemental material. Download AEM.00690-21-s0001.pdf, PDF file, 0.1 MB [file aem.00690-21-s0001.pdf]

```
>putative_BCL_BzlA_Clostridia_bacterium_BF_DJ94058.1
----------------------------MSEINLEIPEKLNIAVDLVDKNIQR-GNGSKVAIYYQDQ--QLTYEDVYKSVNKTGNALK-
SLGIGIEDRVLLLVFDSPEFVAGFFGAIKIGAVPIPTNTMLKPKDYLYLLNDSRAKVAVVS--EALAPLIEEVRSELLYL--RELVVV-GNAGP----
NQLSFAELVAGANDELT--AADTGKDDPAFW-------LYSSGTTGFPKGTVHLQH-DILVACELYGKGIL--NITEN--
DITFSIAKLFFAYGLGNGLYFPFYVGASTVLYPDRFLPDVYFDL----VKKFKPT----LFFGVPTAYGAMLQAADSISDI-DVSS-VRYCVSAGEALP--
KAIFESWKNRFNLII-LDGIGSTEITHI-FISNRPD--DVKPG-TSGKPVPG--YEAKIVDQDG---NKL-PDGELGTLMIK--GDSIAAYYWNK-----HEKTKETFYGPW-
-IITGD-QYYVDPEGYYTYVGRGDDMIKAGGIWVSPVEVESTIMEHPAVLECGVIGAVDQ-DNLIKPKAYVVLK-EGFS------ASEELVT---ELKAFVKER--
IALYKYPRWIEFTQELPKTATGKIMRFMLRKLNEE--------------------------
>putative_BCL_BzlA_Clostridia_bacterium_BF_ADJ94007.1
----------------------------MGEVKTTGVESYNAVTLFVDRHERE-GRGDKVAIYFEDQ--QITYREVREKVNQTGNALK-
ELGIGLEDRVLLLLLDCPEFAYSFFGAMKIGAVPIPTNTILKPADYQYLLNDSRAKAIVVS--EELLGDILEIRSNLKFL--RHIIVV-GKSQQ----
ECISFDDFISGKSTQLE--AEETSKDDPAFW-------LYSSGTTGFPKGTVHLHH-DMAYAAEHYAKGVL--GINEN--
DRTFSVARLFFAYGLGNGLYFSFYVGAATILSANRPTPVNVYEV----IDKYKPT----LFFGVPTSYTAMLQIEGAKEKY-DLSS-IRHCASAGEALP--
RIIFERWRDTYNIEI-LDGIGSTEILHI-YISNMPG--QVKGG-STGKTVPG--YEAKIVDVEG---NVQ-PVNEVGTLQVK--GDSTAAYYWNK-----HEKTKESFKGDW-
-FNTGD-QYYQDEEGYFWYVGRGDDMIKAGGIWVSPIEVENSLLEHPSVLETGVIGSGDE-DGLIKPKAFIVLK-EGYD------PTPELAK---ELSHFVKSK--
IAPYKFPRWIQFVPELPKTATGKIQRFKLRQLEDVG-------------------------
>putative_BCL_BzlA_Clostridia_bacterium_BF_ADJ94003.1
---MIFKMKNVTFVPAHTNFKGGGMMTGGNLII---PENYNAVSFFVDRHLAE-GRGDKTAIYYLDQ--KISYREVAANVNKTGNAFL-
NLGIETEDRILLLLLDCPEFVYSFFGAIKIGAVPIPTNTMLKPTDYEYLLKDSRAKAVVVS--EELIENINEISSNLPSV--EHIIVV-GRSGP----
GQLSFHNLIKNASPVLS--LAPTIKDDPCFW-------LYSSGTTGFPKGAVHLQH-DMVYCAENYAQKVL--NINED--
DVTFSVAKLFFAYGLGNGLYFPFSVGAATVLSPQRPLPEHVFEV----IERYRPS----LFFGVPTSYNALLQLAEEENKY-DLSS-IRSCVSAGETLP--
EIIYQRWMEIFGLEI-LDGIGSTEILHI-FISNRPG--ETKPG-SSGRLVPG--YEAKILDEDG---CEV-PTEEQGTLYIK--GDSIASYYWNK-----HEKSKQTFVGEW-
-INTGD-RYYRDKDGYFWYMGRGDDMIKSGGIWVSLVEVESTLLQHPAVLECAVTGWADS-DGLIKPKAFVVLK-QNSH------ETQALAE---ELQQFVKNK--
IAPYKYPRWLEFIDELPKTATGKIQRYKLRQEG----------------------------
>putative_2-PACL_PITCH v1 a760031 TRIP1_SPD75821.1
-------------------MSQDTRKMFMDVIRKWTPHLDEPATDDCWAPEIEKAGLDKIKEIQSEK-LEVAFRYIYEYSPFYSKKYK-EAGLTPKD----------------
------------------------------------------IKSIDD-LHKIPVTDKEDLRRS-------------------IAACPPWG----
DFSCLDDHYWKQDGWTIWWTTGSTGAPVPCRYTSF-DRVTQAWQAARHMYMSGFRRG--DLAMFCAPFITHMFAWAHLKGLELMKIPAIPAGPPMPTEARIGH----
ILNYRPT----LLLGTPTYMIYLGETIREKGISAKDLS-IREILIGGEPGGSLYATRKRLMNLWGCDV-
CDGFGTTEVGALGGHAHTCVYETKDQGRNSNLHFTEDSGIPEILNPKT---FEPMPDGENGTIVWS--SVSTVSQPILRF----NLNDIMNIQSMD--CPCGR-SFRMAKNG-
--VQGRADDMIHISGVNVFPANIEEAVRSINEFGNEYRLKLMDGKKGMINLVVEVEIM-PEVP----ENDHERLLG---VLQTRIFDK--CQVNPKIESVAY-GSLPR-
AEFKSKRILDLRAK----------------------------
>putative_2-PACL_PITCH v1 a1330026 TRIP1_SPD72413.1
-------------------MRTDSRKMFMDVILKWMPNPLEPAEEDCWAPEMERATPEKIKEIQSEK-LEAAFRYIYEYSPYYSEKYK-KAGLTPKD----------------
------------------------------------------IKSIDD-LHKIPVTNKDDMRKS-------------------IAAHPPWG----
NFSCIDEKLWKTDGWIVFLTTGTSGAPVPVRHTQF-DRVSQSWHLARQWWMSDIKSD--DFVMYCVPFTTHIHAWIHYTAQETARIPMLGAGAPVATEARIDY----
IEKYKPT----VLVGTPTYMIYLGETMKNKGIDPRATS-VRIISCAGEAGGSLLQTRRRLMDLWNADV-
GDLFGTTEAGGVGGHAQMCIYEMKDHGRHGRLHYAEDAGIPEILDPDT---LEPLPEGEFGTLAWS--GVSSIAQPILRF----NIKDIANIKSIE--CGCGR-TMRMSEGG-
```

--IVGRADDMIVIRGVNVFPTNIEEAVRDIDGFGNEYRIKIVEE-RGLPDLIVETEIV-PDVP----EKDHANLVN---ELQAKIKDK--CQIRVSIEVVPF-GSLPR-
SEFKAKRVMDLRETGRFRT-----------------------
>putative_2-PACL_PITCH v1 a1100006 TRIP1_SPD71907.1
------------MSSVKFLFTEENVGKRHGVYPDEEQDRTKPYSEKFWSKVET-LPIERIREIQMER-FRNIVQFAYARSPFYRRIWD-NAGIKPED----------------
--IRGWDD-IRHIPIVTKYDFGDD-------------------QKENPPYGTAFTSPPNTQLKYW--------
QTSGTTAKPRLWTETKE-DWENGIFLYSRGLYAHGIRPG--WRGFFGFSYPPFIAFWLCHSACESMGCQIVPKGP-LSTKAWLGL----
IKNLSTTGVDSFLAATPTFTMRHVEMAEELGINLKELN-IKVLTMAGEPGACVPSTKKYLENAWAAKA-HDQLGSVETSGP--VMYSCAEQAEEENMSDHLNLDS--
FLVELVDPDT---LKPVGDGEPGATVVT--ALTRFGMPTIRF----LLGDWMTISYEK--CRCGR-TLPLAKGG---
IKARSDDLIIIKGTNIYPSLIENSVRSIEGLSPEYRIRVKRT-----NAIVMVEAK-PGIK----KTDYQKLSK---ILEEDIRDK--TSVRLMIEVNPP-GTLPR-
EDVKTKRI-IRE------------------------------
>putative_2-NACL_NaphS2_EFK07530.1
----------------------------------MQLYSDKAYSSPYWNEYLETMPRQQLDQLHLRR-LQKLIKYAYENIPMYGDLYD-KAGVKPED----------------
--IKTLDDFAEKIPLIDKPDLVKY-------------------QSYDPPFGGTIVKDSEDYLTFY-------
FQTSGTTGTPLKEIGYYR-DMLSTGWVF--KWWAHGIRPK--DVFYFAFPFGTFMAFWCAYYDAVALGSQVITAGG-LNTEQRVKQ----IQELKPT----
VLVATPTYAMRIAEVAREMGVDPAQTS-IKYITSAGEQGYVVQTIRDAAEKAWGAKA-IDLYGLSDLWGS--TSWHCP------SNADRMHLTESIAYGLVLDDNG----
NVMPDGGKGEFILT--NY-ATVMPLIKY----RTHDVVEWHKEA--CDCGR-TWLWLRDG---VLGRTDQMVTIKGTNVYPTAIQGIIGNIDGLSEHLEIHFDSG-
EGGDSVSVKVEAE-PDIA----TDKYDALKE---KLSEDLRYR--IGVGMKMEILPP-KSLPR-YELKAKRVFDHREKKK--------------------------
>putative_2-NACL_N47_CBX27264.1
----------------------------------MRLYTQKATESPYWNEYMETMPREKLDQLHLRR-LQRLIKYAYENIPMYQELYD-KAHVKPED----------------
--IKMLDDYVEKIPSIDKPDILRY-------------------QAANPPFGGSIVRDSDEYLTFF-------
FQTSGTTGTPMKEIGYYR-DMLSTGWVF--KWWAHGIRPA--DIFYFAFPFGTFMAFWCAYYDAVAMGAQVITSGG-MTTEQRVRQ----ILELKPT----
VLVATPTYAMRIAEMAREMGVDIAHSS-IKYITSAGEAGYVLPTVRDAVEKAWGAKA-LDLYGISDLWGS--TSWHCP---VHP---DRLHLTETIAYPLVLDKEG----
KLVPDGGIGEWTLT--NY-STVMPLIKY----RTHDTVEWHKET--CECGR-TWTWLRGG---VLGRTDQMVTIKGTNVFPTAIQGIIGGIDGLTENLEIHILTG-
EGGDEIDIKVEAA-PDVP----AEKYEVLKK---HLRGELQFR--IGVKMKVDLLLP-KTLPR-YEVKAKRVFDHRKRK---------------------------
>putative_2NACL_1MN_TDA68259.1
----------------------------------MPLYSEAATKSPYWNEYLETMPRDKLDQLHLRR-LQAMIKYAYENAPMYRDLYD-RAGVKPED----------------
--VKTLDDYIDKIPTIDKSDVVAY-------------------QAKNPPFGDAIVRGGEEYVNIF-------
YMTSGSTGKPMMEPGYFK-DI-HQQWTY--KWWAHGIRQN--DVFYFAFPFGTFMGFWSAYFDALMMGCQVISSGG-QDSKGRIRQ----ILELKPT----
VLCATPTYILHLAEVARDMGVNPAETS-IKFLTMAGEQGAVVASLRKAMEEAWGARA-LDLYGISELWGS--TSWHCP---MH---LDRLHLSETVAYGVVLDEDG---RKV-
PSGGRGEFVLT--TYNATVMPLIKY----RTHDVVEWHLEG--CDCGR-SWLWLREG---VLGRTDQMVTIKGTNVYPAGLQAILGEIPQLSENMEIHFTTE-
ADGDAVTVKVEPI-GNVS----QENYPQLQQ---QVAEELHRK--VGVRIGVELVPP-KSLPR-YEQKAKRIFDNRKK----------------------------
>PCL_Thermodesulfovibrio yellowstonii_ACI20803.1
---MIWNKEFECMSEKKLKALQLER-LKQTVQRAYEKVPYYRKKFD-EVGLKPED----------------
--IKTLDD-IKNIPFTSKADL------------------------REVYPFG--MFASSLSEIVEI-------
HMSSGTTGKPVVAGYTRN-DIDIWAEVMARCLTMAGATKD--DIVQNCYGYGLFTGGFGVHYGAHKIGAMVVPASA-GNTRRQIEI----MRDFGTT----
ILTCTPSYALYMAEVAQEMGIEPTTLK-LKAGCFGAEMWT--EQMRKEIEKRFNLNA-LNIYGLTEIIGP-GVAHECI------E-KKGLHVFEDHFYVEVIDPDT---
GDSLPDGKRGELVLT--TLTREGMPMLRF----RTKDITSLIREK--CSCGR-TFARIER----IRGRTDDMIKVRGVMIFPYQIERTILEVQGVEPHYQIIITRP-
QHLDEIEVMVEMS-KETF-SDEVKHVENLRK---KLEKRIEET--IGIRVKVTLVEP-KSLPR-SEGKAKRVIDKRSLID--------------------------

```
>PCL_Streptomyces_coelicolor_TYP55787.1
------------------------MSSEPTTGTAPAPRRGEPLPHDLLDDAER-LSREQLRELQLDR-LRATLRHAYDNVELYRKKFD-AAGVTPDD----------------
------------------------------------------CRSLAD-LSRFPFTTKADL-----------------------RDTYPFG--MFAVPMADVRRV-------
HASSGTTGRATVVGYTEN-DLSMWADVVARSIRAAGGRPG--HKVHISYGYGLFTGGLGAHYGAERAGCTVIPASG-GMTARQVQI----IQDFRPE----
IIMVTPSYMLTLLDEFERQGVDPRTSS-LQVGIFGAEPWT--EEMRREIEERMDIHA-VDIYGLSEVIGP-GVAQECV--ETK----DGLHIWEDHFYPEVVDPLT---
DAVLPGGEEGEIVFT--SLTKEALPVIRY----RTRDLTRLLPGT--ARP---AFRRMRK----VTGRCDDMIILRGVNVFPTQVEEIVLRTPGVAPHFQMRLTER-
GRMDHMTVRVEAR-PDAA----PEQRDAAAR---AIAQGVKDG--VGVTVEVEVVEP-ETLER-SLGKIRRVWDQRGA----------------------------
>PCL_Rhodospirillum rubrum ATCC 11170_ABC24298.1
-----------------------------------------MVRFFDALETREPEVRDREQAAALPG----LIQHAKDHAPAYARLLA---DIDPP-----------------
------------------------------------------AITSREALAALPVTRKSALISL-------------------QAEAPPFG--GFATSRGAFSRV-------
LQSPGPIYEPEGKRSDY--------WRLSRALFAAGFRPG--DLVHNCFAYHFTPGGWIFDDGAQALGCPVFPAGT-GQTEQQLQA----IAALRPT----
AYVGTPSFLAILLDKADYLGA--DISS-LKKGLVSAEAYL--PPQRTLFAER-GIPT-YQCYSTADLG---LIAYESP---DDSGAVEGMVVDEG-VILEIVRPGT---
GDLVAPGEVGEVLVT--TFTPE-YPLIRF----ATGDLSATLPGP--SPCGR-TNMRIKG----WMGRADQTTKVKGMFVHPQQVADVVRRHDALAKARLI-VTRI-
EGGDVMTLVCETT-----------ETLDLAD---QVAESLRVV--TKLRGEVRFVTP-GSLPN--DGKV--IEDARDYG---------------------------
>PCL_Pseudomonas putida_WP_059394932.1*
------------------------------------MNMYHDADRAL-LDPMETASVDALRQHQLER-LRWSLKHAYDNVPLYRQRFA-ECGAHPDD----------------
------------------------------------------LTCLED-LAKFPFTGKNDL-----------------------RDNYPYG--MFAVPQEEVVRL-------
HASSGTTGKPTVVGYTQN-DINTWANVVARSIRAAGGRKG--DKVHVSYGYGLFTGGLGAHYGAERLGCTVIPMSG-GQTEKQVQL----IRDFQPD----
IIMVTPSYMLNLADEIERQGIDPHDLK-LRLGIFGAEPWT--DELRRSIEQRLGINA-LDIYGLSEIMGP-GVAMECI--ETK----DGPTIWEDHFYPEIIDPVT---
GEVLPDGQLGELVFT--SLSKEALPMVRY----RTRDLTRLLPGT--ARPMR-RIGK-------ITGRSDDMLIIRGVNVFPTQIEEQVLKIKQLSEMYEIHLYRN-
GNLDSVEVHVELR-AECQ-HLDEGQRKLVIG---ELSKQIKTY--IGISTQVHLQAC-GTLKR-SEGKACHVYDKRLAS---------------------------
>PCL_Paak2 Burkholderia cenocepacia J2315_2Y4O_A*
-----------------------------------GSHMTHPTHPAAALEPIETASRDELQALQLER-LKWSLRHAYDNVPHYRRTFD-AAGVHPDD----------------
------------------------------------------LKSLAD-LAKFPFSTKNDL-----------------------RDNYPFG--LFAVPREQVVRV-------
HASSGTTGKPTVVGYTAR-DIDTWANVTARSIRAAGGRPG--DTLHNAFGYGLFTGGLGIHYGAERLGCMVVPMSG-GQTEKQVQL----IRDFEPK----
IILVTPSYMLNLIDEMVRQGMDPAESS-LKIGIFGAEPWT--QALRNEVETRVGIDA-LDIYGLSEVMGP-GVACECV--ETK----DGPVIWEDHFYPEIIDPVT---
GEVLPDGSQGELVFT--SLTKEAMPVIRY----RTRDLTALLPPT--ARAMR-RLAK-------ITGRSDDMLIVRGVNVFPSQIEEIVVALPLLSGQFQITLSRD-
GHMDRLDLAVELR-SEAAASVTDGERAALAR---ELQHRIKTM--VGVSSGVTVLAA-GGIPATATGKARRVIDRRQAA---------------------------
>PCL_Paak1 Burkholderia cenocepacia J2315_2Y4N_A*
-------------------------------------GSHMASTTPLPLEPIETASRDELTALQLER-LKWSLRHAYDHSPVYRRKFD-EAGVHPDD----------------
------------------------------------------LKTLAD-LSRFPFTTKGDL-----------------------RDSYPFG--MFAVPQDRISRI-------
HASSGTTGKPTVVGYTAA-DIDTWANLVARSIRAAGARRG--DKVHVSYGYGLFTGGLGAHYGAERAGLTVIPFGG-GQTEKQVQL----IQDFRPD----
IIMVTPSYMLSIADEIERQGLDPVQSS-LRIGIFGAEPWT--NDMRVAIEQRMGIDA-VDIYGLSEVMGP-GVASECV--ETK----DGPTIWEDHFYPEIIDPET---
GEVLPDGELGELVFT--SLTKEALPIIRY----RTRDLTRLLPGT--ARTMR-RMEK-------ITGRSDDMMIVRGVNVFPTQIEEQLLKQRALAPHYQIVLTKE-
GPLDVLTLNVEPC-PETA---PDTAAIQVAK--QALAYDIKSL--IGVTAVINVLPV-NGIER-SVGKARRVVDKRKG----------------------------
>PCL_Methanothermobacter thermautotrophicus_WP_010877457.1
---------------------------------------------MIWNPEAECMSQEEKQELQLRR-LQNTVKRAYENVPYYNKRLR-DAGVFPED----------------
------------------------------------------IETLDD-IEKLPFTTKNDL-----------------------REAYPFG--MFAVPDEEIVEV-------
```

HTSSGTTGKPVVSGYTSR-DLEIWSEVMARALTMGMATRK--DRIQNCYGYGLFTGGLGVHYGAQKIGATVIPISA-GNTKRQIEI----MQDFGTT----
VITCTPSYALYLAEVLEKEGVDIGELN-LKSGIFGAEMWT--EEMRETIEARLGLTA-LNIYGLTEIIGP-GVAMECT------E-KNGLHIAEDHFYPEIIDPKT---
GEKLPNGTKGELVLT--TLTREGMPVLRF----RTKDITALRDGE--CGCGR-TLVRMDR----ITGRSDDMLKIRGVIVFPSQIERALLKIKGLEPHYQIVVTRP-
EFLDELEVQVEAS-PELF-SDEVKHVEEAKR---MIEKHIHSE--IGLRVNVTLVEP-GSLPR-SEGKAIRVIDKRKFD---------------------------
>PCL_Geobacter sulfurreducens_AAR35114.1
--MSYHNEEFETLPRQALEALQLKR-LQATVARVQASVPFYRQSFE-RAGIISGC----------------
--IKSLDD-LRRLPFTVKQDM-----------------------RDSYPYG--LFAAPMDDIVRI-------
HASSGTTGKPTVVGYTRK-DIEIWSELMARSFAAAGVHKG--DIIHNAYGYGLFTGGLGAHYGAERLGASVIPMSG-GNTKKQIMI----MKDFGST----
VLTCTPSYSLFMAEAAREEGVDFRQLK-LHVGIFGAEPWS--ESMRAEIEQKLNLCA-IDIYGLSEIMGP-GVAIECR--EAK----KGLHIWEDHFIPEIINPET---
GEVLPEGERGELVIT--TITKEGIPLIRY----RTRDITSLTYEP--CSCGR-THARLSR----MTGRSDDMLIIRGVNVFPSQIESILMRIEGVEPHYLLIIDRK-
DNLDTLEVQVEVD-EQLF-SDEIKVLQALSH---RIEKEIKDL--LGVTCKVRLVEP-QTIAR-SEGKAKRVIDNRLIS---------------------------
>PCL_Deinococcus radiodurans R1_AAF12384.1
--MFQPEREALPLPQLRALQLAQ-LQVMVARQYERVPAYRAKFD-ALGVRPDD----------------
--LQTLDD-LARFPLTRKSDL-----------------------RENYPLG--LLATDRDQLRRI-------
HASSGTTGKPTVVAYDAN-DLNVFSDVVARSLYAAGGRPG--MTFHNAYGYGLFTGGLGTHGGAERLGMCTVPISG-GGTEKQVQL----IQDLEPQ----
IIACTPSYALVLAEALGRCGMRPEDIS-LQYAVLGAEPWS--NKTRTEVEARLGVKA-TNIYGLSEIIGP-GVSNEDV---SEQ---RGSYLWEDHFYPEILDPDT---
GEVLPDGEWGVLVLS--SMTRTALPVLRY----WTGDITRLLPAQ--NGTGR-TMRRMDQ----IRGRSDDLIILRGVNVYPTQLEAVLLGMGQASPHYHVILTRT-
GIMDDLTLQIEAL-SESA---------ALRQ---EIERQIKAQ--VGVTVRCELHVP-GSLPRSEGGKLRRVTDLRGDR---------------------------
>PCL_Bradyrhizobium diazoefficiens USDA_BAC48162.1
-------------MVAGTRPIKKRTDQCGETRMALTRLKEGGTSYRAEMDAHERASRDEIMALQKQR-LGWSLKHAYDNVAHYRKAFD-KAGVHPSD----------------
--FRELSD-LAKFPFTVKTDL-----------------------RDNYPFN--MFAVPREKLVRV-------
HASSGTTGKPIVVGYTQR-DIDTWSEVMARSIRAAGGRTG--MIIHNAYGYGLFTGGLGVHYGAEKLGCTVVPISG-GMTERQVQL----INDFRPD----
IITVTPSYMLAILDEFKRQKLDPRQCS-LKVGIFGAEPWT--NAMRGEIEDAFDMDA-TDIYGLSEVIGP-GVAQECI--ETK----DGLHIWEDHFYPEVIDPET---
GAVLPDGEKGELVFT--SLTKEGFPVIRY----RTRDLTRLLPGT--ARPGM---RRMEK----VTGRSDDMIILRGVNLFPTQIEEVLLATDWCGGHFILELTRE-
GRMDELTIIAEAR-PESW------DGRGLVDHADRISTHIKNT--IGISSNVRVVAP-ATLER-SLGKARRLYDKRPKD---------------------------
>PCL_(anaerobic)_Azoarcus evansii_CAD21694.1*
-----------------------------MSARDGFAVPKGAARDTL--DPIETASRDELAALQLER-LKWSLQHAYDNVAHYRQAFD-EAGVHPAD----------------
--LKQLSD-LSKFPFTTKKEL-----------------------RDNYPYG--LFAVPMRDIVRV-------
HASSGTTGQPTVVGYTKK-DIAMWGTVMARSLRAAGGTPE--DIILNSYGYGLFTGGLGAHYGGERLGATVIPMGG-GNTEKQIQL----IREFKPT----
MMMATPSYMLTVADGLQEMGIDPASTT-LRVGVFGAEPWT--NEMRREIETRLGIDA-IDIYGLSEVIGP-GVACECI--ETK----DGPHIWEDHFYPEIIDPVT---
GEVLPDGTPGELVFT--SLTKEALPIVRY----RTRDLTVLLPGT--ARTMR-RIGK-------ITGRSDDMLIIRGVNVFPTQIEEILLRHDSLCGHYQLQITRP-
GHMDELTVLAEIR-HDLSDSVNEAQRSKIAA---EVRHEIKSR--VGVSADVQIVET-GRIERTQVGKAKRVIDKRPKE---------------------------
>PCL_(aerobic)_Azoarcus evansii_WP_169132234.1*
--------------------------------------MPVKTPSPGDLEPIEKASQDELRALQLER-LKWSVRHAYENVPHYRKAFD-AKGVHPDD----------------
--LKSLAD-LAKFPFTAKGDL-----------------------RDNYPFG--MFAVPREKVARV-------
HASSGTTGKPTVVGYTLK-DIDTWATVVARSIRASGGRAG--DMVHIAYGYGLFTGGLGAHYGAEKLGCTVVPMSG-GQTEKQIQL----IQDFKPD----
IIMVTPSYMLTVLDEMERMGIDPHQTS-LKVGIFGAEPWT--QAMRAAMEARAGIDA-VDIYGLSEVMGP-GVANECI--EAK----DGPVIWEDHFYPEIIDPHT---

```
GEVLPDGSEGELVFT--TLTKEAMPVIRY----RTRDLTRLLPPT--ARSMR-RMAK-------ITGRSDDMLIIRGVNLFPTQVEELICKNPKLAPQYLLEVDKD-
GHMDTLTVKVEIN-PEANVGRHPEQKEALAK---ELQHDIKTF--IGVSAKVHVCEP-FAIERVTIGKAKRVVDRRPKE---------------------------
>BCL_Thauera aromatica_AAN32623.1*
---------------MYTLSVADHSNTPPAIKI---PERYNAADDLIGRNLLA-GRGGKTVYIDDAG--
SYTYDELALRVNRCGSALRTTLGLQPKDRVLVCVLDGIDFPTTFLGAIKGGVVPIAINTLLTESDYEYMLTDSAARVAVVS--QELLPLFAPMLGKVPTL--EHLVVA-G--
GA----GEDSLAALLATGSEQFE--AAPTRPDDHCFW-------LYSSGSTGAPKGTVHIHS-DLIHTAELYARPIL--GIREG--
DVVFSAAKLFFAYGLGNGLIFPLAVGATAVLMAERPTPAAVFER----LRRHQPD----IFYGVPTLYASMLANPDCPKE--GELR-LRACTSAGEALP--
EDVGRRWQARFGVDI-LDGIGSTEMLHI-FLSNRAG--DVHYG-TSGKPVPG--YRLRLIDEDG---AEITTAGVAGELQIS--GPSSAVMYWNN-----PEKTAATFMGEW-
-TRSGD-KYLVNDEGYYVYAGRSDDMLKVSGIYVSPIEVESALIAHEAVLEAAVVGWEDE-DHLIKPKAFIVLK-PGYG------AGEALRT---DLKAHVKNL--
LAPYKYPRWIEFVDDLPKTATGKIQRFKLRSA-----------------------------
>BCL_Paraburkholderia xenovorans LB400_AIP34616.1
-----------------MEALLEKAANPPAATVEAPPALFNFAAYLFRLNE---TRAGKTAYIDDTG--STTYGELEERARRFASALR-
TLGVHPEERILLVMLDTVALPVAFLGALYAGVVPVVANTLLTPADYVYMLTHSHARAVIAS--GALVQNVTQALESAEHDGCQLIVSQPRESEP---
RLAPLFEELIDAAAPAAK--AAATGCDDIAFW-------LYSSGSTGKPKGTVHTHA-NLYWTAELYAKPIL--GIAEN--
DVVFSAAKLFFAYGLGNGLTFPLSVGATAILMAERPTADAIFAR----LVEHRPT----VFYGVPTLYANMLVSPNLPAR--ADVA-IRICTSAGEALP--
REIGERFTAHFGCEI-LDGIGSTEMLHI-FLSNRAG--AVEYG-TTGRPVPG--YEIELRDEAG---HAV-PDGEVGDLYIK--GPSAAVMYWNN-----REKSRATFLGEW-
-IRSGD-KYCRLPNGCYVYAGRSDDMLKVSGQYVSPVEVEMVLVQHDAVLEAAVVGV-DH-GGLVKTRAFVVLK-REFA------PSEILAE---ELKAFVKDR--
LAPHKYPRDIVFVDDLPKTATGKIQRFKLREQ-----------------------------
>BCL_Magnetospirillum sp. TS-6_BAE91925.1*
-------------------MTSPNASSEHALDI---PRDYNAATWFIDRHLKD-GRADKVAFIDADG--SHTYGQLADKVNRAGNALK-
GLGLHMENRIAMIMLDTVDFPAVFWGAVKAGIVPIPLNTLLTTGDYGYMLSDSRARVLVIS--EELFDKVEPILPDLPML--EHVVIS-GKNAH----
GHTLLSDLLAKAEPKLK--TAETTRDDVAFW-------LYSSGSTGAPKGAVHLQR-DLPATAVHYGQQVL--GIRED--
DVTYSAAKLFFAYGLGNGMTFSLHVGATSVLLKDRPTPEAVMKL----LKDHQPT----IFYGVPTLYGTILADPQYRRET-ASTR-LRACVSAGEALP--
EDVGRRWEERFGAAI-LDGLGSTEMLHI-FLSNRHG--EVRYG-TSGKPVPG--YELKICSDDG---HEV-PQGEMGELVVR--GPSSATAYWNQ-----REKSLKTFRGEW-
-THTGD-KYYVDDDGYYRYAGRGDDMLKVGGIWVSPFEVEAALISHDKVLEAAVVGETDA-EGLVKPKAFVVLA-PGET------GSEVLKE---ELQAYVKSK--
LAPYKYPRWVEFVEALPKTATGKIQRFKLRGTLSQK-------------------------
>BCL_Azoarcus evansii_CAD21640.1*
---------------MAELSVADHSVSPPRITI---PREYNAAHDLIERNLRA-GRGGKTAVIDHAG--SYTYAQLAERVDRFAHALG-
ELGIRMEERVLLCLLDTIDFPTAFLGCIKAGVVPIPVNTLLTASDYTYMLRDSRARGLVVS--SALLPAFSNAIEASPFI--KNVMVSGGDAGT--
RGGHLDFAELIASPRPPYE--AAQTCCDDPCFW-------LYSSGSTGAPKGTVHLHS-SLIHTAELYANAIL--GVRED--
DVVFSAAKLFFAYGLGNGLTFPLSAGATAVLMAERPTPDAVFRV----LREHQPT----IYCGVPTLYASMLASPALPGR--EELS-IRRCASAGEALP--
AEVGNRWTEHFGVEI-LDGLGSTEMLHI-FLSNRAG--DVHYG-TSGKPVPG--YELRLIGDDG---EEVAP-GEAGELQVR--GPTSAALYWNN-----RTKSRETFVGQW-
-TRSGD-KYSQDADGNYVYAGRNDDMLKVGGIYVSPIEVESALITHAAVLEAAVVGKADD-DGLIKPLAFVVLK-PGRM------PAAELAD---ELKLHVKSK--
LAPYKYPRWLEFVDELPKTATGKIQRFKLRSLSGA--------------------------
>BCL_Azoarcus evansii_AAN39371.1*
---------------MTTLSAADHSTSPPTITL---PRQYNAADDLIGRNLDA-GRGSKIAYIDDNG--RYSYDELAARVNRFANALG-
ALGITREQRILMCVHDTIDFPTVFLGAIKAGVVPIAVNTLLTQSDYEYMLSDSRARIAVVS--APLYDTFAPLLGKVESL--ERIVVA-G--GE----
GPDSVAALMANASDKFE--AVATTCDDPCFW-------LYSSGSTGAPKGTVHIQS-SLIHTAELYAKPIL--GIKES--
```

DVVYSAAKLFFAYGLGNGLTFPLSVGATAVLMAERPTPAAAFKR----LREQQPT----IFYGVPTLYASMLADADCPTR--EELA-IRMCTSAGEALP--
EDIGRRWTERFGVEI-LDGIGSTEMLHI-FLSNRPG--DVHYG-TTGKPVPG--YQVRLVDDEG---NVVEGADEPGELQIS--GPTSAVMYWNN-----REKTRATFQGPW-
-TRSGD-KYSRNAEGYYVYAGRNDDMLKVSGIYVSPIEVESCLIQHPAILEAAVVGHEDE-ERLIKPKAFIVLK-PGFQ------RSEQLAA---EIKAHVKAH--
LAPYKYPRWMEFVDELPKTATGKIQRFKLRAMAGK--------------------------
>BCL_(badA)_Rhodopseudomonas palustris_L42322.1*
---------------------------MNAAAVTPPPEKFNFAEHLLQTNR---VRPDKTAFVDDIS--SLSFAQLEAQTRQLAAALR-
AIGVKREERVLLLMLDGTDWPVAFLGAIYAGIVPVAVNTLLTADDYAYMLEHSRAQAVLVS--GALHPVLKAALTKSDHEVQRVIVSRPALRWS------
RARSTSLSRRTSLEK--PAATQADDPAFW-------LYSSGSTGRPKGVVHTHA-NPYWTSELYGRNTL--HLRED--
DVCFSAAKLFFAYGLGNALTFPMTVGATTLLMGERPTPDAVFKRWLGGVGGVKPT----VFYGAPTGYAGMLAAPNLPSR--DQVA-LRLASSAGEALP--
AEIGQRFQRHFGLDI-VDGIGSTEMLHI-FLSNLPD--RVRYG-TTGWPVPG--YQIELRGDGG---GPV-ADGEPGDLYIH--GPSSATMYWGN-----RAKSRDTFQGGW-
-TKSGD-KYVRNDDGSYTYAGRTDDMLKVSGIYVSPFEIEATLVQHPGVLEAAVVGVADE-HGLTKPKAYVVPR-----------PGQTLSE--TELKTFIKDR--
LAPYKYPRSTVFVAELPKTATGKIQRFKLREGVLG--------------------------
>4-HBCL_Gemmatimonadetes bacterium_OLC05270.1
------------------------------MDI---PHDFNAATWFVDRHVAE-GRAKRVAIRYDGQ--DITYGDVLAGANRCGNALR-
RIGVRMEQRVLLLLPDCPEFVYAFWGALKIGAVPIPTNTLLKPRDYEYILNDSRAEVAVVG--EELAPAIEEVRPRLRFL--HQLVVA-GRAGP----
GQVGLSELVAEEPADLD--AAATTKDDIAFW-------LYTSGTTGMPKAAVHLQH-DMLVCCEGYGRHVL--GIGPD--
DRCFSVARLFFAYGLGNALYFPFHVGASTVLFPRRPEPAKAFEI----LTRERPT----LFFAVPTAFAAMLQVPNAERTY-DLRS-VRLCLSAGEPLP--
KPIYERWLERFRLEI-LDGIGSTELCHT-FIANRRG--HVRPG-SSGTLVPG--YDARIVDDDG---RDV-PPGEVGNLLVQ--GDSACAGYWNQ-----HERSKQTFVGEW-
-VRTGD-KYRRDPDGCFWYAGRADDMIKAGGMWVSPAEVESALAEHPAVVEAGVVGAAGR-DELVKPLAFVVLA-PGHA------PSPALED---ELKGFVKQR--
LAVYKYPRWIVFVPELPKTATGKIQRFRLREIAAQTHSTSKG-------------------
>4-HBCL_Desulfobulbus sp._KGO35804.1
--------------------------MHVELNL---PETFNAADYFVDRNIRE-GRGQKTAILCRDN--AFTYAQVQAGMNKVGNALK-
GLGIEMENRVALLLLDSEFYPLSFFGAIKIGAVPICLNTLMQPKDYLYFLNDSRAKILIID--EELYSKFEPIKDKLRFL--KHIIVVNGQAPA----
GTISFDSFVAGASDQLE--AAPITPDDACFW-------LYSSGSTGQPKGTIHLQH-DMVYAAETYGKQVL--GIQEH--
DICFSAAKLFFAYGLGNGLYFPFSVGATSVLMPVRPVPASVYET----IARYRPT----LYFGVPTLYGSMLQEEGS------LDG-VRLCVSAGEALP--
ADIFKRWQQRHGVEI-IDGIGSTELSHI-FISNLPG--KAKPG-SSGKVVPG--YDARIVDENF---QDI-PVGEVGTLLIK--GDSAAAAYWNK-----HQKTKSTMLGDW-
-VNTDD-KYAVDEEGYFYYIGRSNDMLKVGGIWVSPVEVEACLIEHPAVLECAVVGATDD-KNLVKPKAFVVLK-RGYA------PDDQLKK---DLKEYVKKT--
LAHYKYPRWIEFVDDLPKTATGKLKRFELRA------------------------------
>4-HBCL_Candidatus Rokubacteria bacterium_OLB51045.1
-----------------------------MNASDFLPAQFNAAAWFVDKNVAE-GRGAKPAFLYEDR--TLTYADAHELVNRTGNALR-
ELGVEMEHRVLMLCLDAPEFIGTFWGAIKIGAVPVPVNTLMRTTDYRYFLDDSRARVAVVS--APLLAEAAPALVEAKHL--KHVLIAGGSHGP-----
FMSYEDRIARASATLE--AAPTSRDDAAFW-------QYSSGSTGFPKGAVHLHH-DMAVCCETYGKQVL--GIQPT--
DRVFSAAKLFFAYGLGNACFFPMSVGAQSVLYPHRPTPESVFEI----IARYRPT----LFFGVPTLYAGMLAVKEAEARF-DTAS-LRLCVSAGEALP--
DAIYTRWRERFRVEI-IDGIGTTEIGHI-FISNRPG--LARPG-SSGLPVPG--YELAIVDDEG---RSV-PSGEIGNLRVK--GDSTMAYYWNK-----HEKTKATLFGAW-
-IQTGD-KYYADEDGYFWYCGRSDDMLKVGGIWVSPVEVEATLVRHPAVLEAAVVGQEDT-DRLVKPKAVIVLK-EPWK------ASEELAN---ELKTFVKDK--
IAPYKYPRWIEFAGELPKTATGKIQRFKLRR------------------------------
>4-HBCL/BCL_Thauera aromatica K172_AVR88075.1*

---------------MPTLSAADHTASPPEIRI---PRHYNAADDLIGRNLDA-GRGPKIAYIDDEG--
RYSFDELARRVNRCGSALGEVLGLRREERVLMCVHDTIDFPTVFLGAIRAGIVPIAVNTLLTASDYEYMLTDSRARVAIVS--EPLMAVFGPLLGKVPTL--ERIIVA-
GDPAA----GADSLAALLAQGREAFP--AAPTVADDACFW-------LYSSGSTGAPKGTVHIHS-SLIHTAELYARPVL--GIRED--
DVVFSAAKLFFAYGLGNGLTFPLAVGATAVLMAERPTPAAVFER----LRQHRPT----IFYGVPTLYASMLASPDCPAR--GELN-LRACTSAGEALP--
EEIGRRWTERYGLDI-LDGIGSTEMLHI-FLSNRPG--QVRYG-TSGMPVPG--YRVRLIDDAG---NEITAPGEPGELQIS--GPTSAVCYWNN-----REKSRSTFLGEW-
-TRSGD-KYLLDQDGYYVYAGRSDDMLKVSGIYVSPIEVESALIAHEAVLEAAVVGCEDE-DHLIKPKAFIVLK-PGLE------ADEALRE---MLKNHVKAM--
LAPYKYPRWMEFVEELPKTATGKIQRFKLRGMGAR-------------------------
>4-HBCL/BCL_Rhodopseudomonas palustris_Q53005.1*
---------------------------MP-------LRDYNAAVDFVDRNVAE-GRGGKIAFIDPQR--SLSYGELRDAVARVGPMLA-
RLGVEQENRIALVLKDTVDFPILFWGAIRAGIVPVLLNTRLTADQYRYLLEDSRSRVVFAS--SEFLPVIEEAAADLPHL--RTIIAV-GDAPA----
PTLQLANLLATEQEGGA--PAATCADDIAYW-------QYSSGTTGMPKGVMHVHS-SPRVMAENAGRRI---GYRED--
DVVFSAAKLFFAYGLGNAMFCPMGIGATSVLYPERPTADSVFDT----LRLHQPT----LLFAVPTLYAAMLADPRSRTET-LPDR-LRLCVSAGEPLP--
AQVGLNWRNRFGHDI-VNGVGSTEMGHL-FLTNLPH--AVEYG-TSGVPVDG--YRLRLVGDRG---QDV-ADDEIGELLVS--GGSSAAGYWNQ-----RDKTRTTFVGEW-
-TRTGD-KYHRRADGVYTYCGRTDDIFKVSGIWVSPFEIEQALMSHAKVLEAAVIPAEDT-DGLIKPKAFIVLASRGDI------DPGALFD---ELKEHVKSA--
IGPWKYPRWIQIMDDLPKTSSGKLQRYLLREMTLGGIEATESAPSEPALYGRVVAGNGR--
>4-CBCL_Thermogemmatispora argillosa_BBH92115.1
------------------------------MSDIPLPLSGTPMTLPTLVELAVSRTPDQLALVEQEQ--HYTYAQLYSQVCAQGNALS-
EAGVAPGERVITVLPTSASHALLLLAIFQIGAVAVPINPRLKPDEVAYCIEHAQARAVCFE--SRTAPVVLEAVRKLAASWRPRLLAVGGEISAPEVTPLDVLARQARRERP-
----AVTIDPTDLSLI-------LYTSGTTGRPKGVTITHA-ASVARVMGLALNHGL-TALAG--LRVLGLMPLFHTVGLHGVFLTALALNGTYYPLAE-FQPARALQL----
IQDAQID----YLFGSPTHFHALLSHPASQEV--DLSS-LKHLLYAGAPMP--TPLLLQCSQRLCSNL-THIYGNTETYNSLFYRHAAE--------
APQALVPGVYHRVRVVSIGGAPEDEV-PAEVEGELIVDTHSPESFSGYWRN-----PEATARCVRDGW--YYTGD-
VCLRDRAGRFFIVGRVDDMIISGAENIHPAEVEEALLSHPGVGDAGVVGVPDP-HWGQRVVAFIERRDPALT-----------AE---
ELDQHCRQHPTLATFKRPRQYVFVEKLPRNPSGKLLRFLLREQAAQSGAVSERELLP---------------
>4-CBCL_Pseudomonas sp. CBS3_ABQ44579.1*
---------------------------------------MQTVHEMLRRAVSR--VPHRWAIVDAAR-STFDICRTGETSRNEGSATA-
RLWPQPARPLAVVSGNSVEAVIAVLALHRLQAVPALMNPRLKPAEISELVARGEMARAVVANDAGVMEAIRTRVPSVCVLALDDLVSGSRVPEV------------
AGKSLP----PPPCEPEQAGFV-------FYTSGTTGLPKGAVIPQR-AAESRVLFMATQA---GLRHGSHNVVLGLMPLYHTIGFFAVLVAAMAFDGTYVVVEE-
FDAGNVLKL----IERERVT----AMFATPTHLDALTTAVEQAGA--RLES-LEHVTFAGATMP--DTVLERVNRFIPGEK-VNIYGTTEAMNSLYMRAVRI--------
AGTVMRPGFFSEVRIVRVGG-DVDDGCPTVKRASWRWR-----RRMRPFQA-----
TLTNLRLLQKSFRKAGTGRAICVRDGSGNIVVLGRVDDMIISGGENIHPSEVERILAAAPGVAEVVVIGVKDE-RWGQSVVACVVLQ-PGAS---------ASAE---
RLDAFCRAS-ALADFKRPRRYVFLDELPKSAMNKVLRRQLMQHVSATSSAAVVPAPAVKQRTYAPSGRAIAR
>4-CBCL_Bradyrhizobium diazoefficiens USDA110_NP_770176.1
---------------------------------------MRRPMLDLASSFMASAARDPNAIALVDGDLRLTYRQWYDKISALVASFD-
RLGLKPGDHVVTLLQNRWEAATLHWACQIAGLVITPINWRAKADELDYCIENAEA-----------
CAVFHQDISAEPVQGSALAGRLLRVSVDPGTGEATSFSDLIKDRAPDAE---PRVGADAWSIM-------LYTSGTTSRPKGVPRRHRAERAAAIAHVAQNLYGRG------
ERTLGVMPLYHTMGVRSLLAMSL-IGGTFICLPR-YDSRQALAL----IEKEEIT----NLYLVPTLYHDLVHHETFAGT--NVSS-VRKLGFAGASMT--
DGLLKKLNEAFKPNLFVNHYGSSEIYTF-TIDQNAA---AKPG-SAGKAGLN--QHVKVVRIGARSVAELAAVGEEGEIIATLAGDEAFEGYWRR-----PEADAKSLREGW-

-YFTGD-TGYVDPDGDLFVTGRVDDMIITGGENVSPVEIESCLSLHPDVDEVAVVGVADE-KWGKIVAAFVKRN-RNVS-----------ES---ELEQFCRTS-
GLANFKRPRRYVFVDAIPKSPVGKLLRRLLVAGEYETERRPPSDAA----------------
>4-CBCL_Alcaligenes sp. AL3007_AAN10109.1
---------------------------------------MQTVNEML-RRAATRAPDHCALAVPARG-LRLTHAELRARVEAVAARLH-
ADGLRPQQRVAVVAPNSADVVIAILALHRLGAVPALLNPRLKSAELAELIKRGEMTAAVIAVGRQVADAIFQSGSGARIIFLGDLVRD-GEP---------------
YSYGPPIE--DPQREPAQPAFI-------FYTSGTTGLPKAAIIPQR-AAESRVLFMSTQV---GLRHGRHNVVLGLMPLYHVVGFFAVLVAALALDGTYVVVEE-
FRPVDALQL----VQQEQVT----SLFATPTHLDALAAAAAHAGSSLKLDS-LRHVTFAGATMP--DAVLETVHQHLPGEK-VNIYGTTEAMNS-LYMRQPK--------
TGTEMAPGFFSEVRIVRIGG-GVDEIVANGEEGELIVA-ASDSAFVGYLNQ-----PQATAEKLQDGW--YRTSD-
VAVWTPEGTVRILGRVDDMIISGGENIHPSEIERVLGTAPGVTEVVVIGLADQ-RWGQSVTACVVPR-----------LGETLSA--DALDTFCRSS-
ELADFKRPKRYFILDQLPKNALNKVLRRQLVQQVSS--------------------------
>3-HBCL_Thauera aromatic K172_AVR88779.1*
--------------------------MSEQLQP---QQSMNAADEIIGRPLAQ-GLGEQTAMLCAER--SITYRELDAATNRHGNALR-
AHGVGKGDRVLFLMDDSPELVAAYLGTLRIGAVAVALNVRLAPRDVLYVIQDSACRLLYID--AEFLHLYQQIAGELEQP--PQVVVR-GDEAP--
APAIIAFKHFLDGQAATLE--SVQVAPDDVAYW-------LYSSGTTGRPKAVMHAHR--SVLIADRLEREYF--GIKPG--
DRVFTTSKMFFGWSLGHSLMGGLQCGATVIVAPGWPDAERVMAT----AARHRPT----ILFSTPVMYRNLLREGAGESA--AMRD-IRHFVSAGEKLP--
ENIGQQWLDTFGIPI-TEGIGASETVFL-FLCARPD--AYRIG-SCGKRVPW--AEVRLLDELG---NEITTPDTPGLIAIR--MASQFVGYWKL-----PETTEKALRDGW-
-YYPGD-MFSFDADGFWYHNGRADDMLKISGQWVSPGEIESCASAVPGIAEAVVVAVPND-DGLTRLTLFIVPE-DPSA------SQQKLSE---AVMTTLRGT--
LSIYKCPRTIQFLEELPRTATGKVQKYRLRDMLQATL------------------------
>2-ABCL_Clostridia bacterium BF_ADJ93975.1
MNGIKLFENVPERFLPPKEDWPDFIYAHPDVNWVYYRDYVNATEEILDVGIAKNGWGNNIAIYDANKNETWTYNRLQEEVNRFGNALK-
KLGVQPGDRVMWRFGEVPYAAVAELAIWKIGAINVGSALQERAREVEFIANDTEAVMIICQ--ADQTDQVQKALPNLKTV--KSVIAVPESLDD----
NFLDYSKLLSEASAELE--PYPTKPGDAASI-------FYTGGTTGHAKGCVHPHA-SEVIIADMLGKVAY--GLTEK--DVMLCSPPVGHSFGNGEKVNYPFRHGASSV-
YCDRPTPQDYWDL----LTTYKVS----IFIGVPTLYRVLLESYKPEYKA-GISN-LRLCVAAGEMLT--NDLYEKWQEQIGVSM-NNSVGMTPMRHS-
FLESLRDGKKVAPGVSAGKLLPG--YEYRLIDEDG---NTV-KNGETGRMAVR--GLTGIV-YWNNLHPAMPGKQKEDVRSGW--NWLDD-
AYVQDKDGWLYFSTRLDNMIVTGGRQIAAPEVEEVLGEHPAVNQVAVVGVPDP-VRTQSVKAFVVLN-SGYK------PSEEMVK---ELQTYAKDQ--
MSFYKYPRIVEFIDELPRDHLGKIQRRVLRERSS---------------------------
>2-ABCL_Azoarcus evansii_AAL02077.1*
--MTSHVDTFARDRLPPPEQQPEFLFELPSLQF---PPLMNCAVELLDRRVLG-GEGERVCLRAPGG-
LRWTYRDLLGHANRIANVLVHELGVVPGNRVLLRGPNSPMLAACWFAIMKAGAIAVATMPLLRAKELGQILDKGRITHALCA--HALRGELDEAVATRPSV--AHVVSF-
GDPAG------AGLEAAMARQSGEFD--NVATASDDTCIL-------AFTSGTTGQPKATMHFHR-DVIAACRCWPPHVL--RPQPD--
DVFIGSPPLAFTFGLGGMLLFPMSVGASTVLLEQ-ASPPKLLDA----IGEFGAT----ILFTAPTSYRAMAEGARERRLGAPLGGPLVKCVSAGEVLP--
AATRALWKDATGIEI-IDGIGATEMFHI-FISADEE--HARPG-ATGTVVPG--YRARIVDDEG---REV-PAGTVGRLAVK--GPTGCRYLD-------DSRQRNYVGDGW-
-NYTGD-AYYMDADGYFHYQSRLDDMIVSAGYNIGAPEVEDALMQHPAVAECAVIGVPDE-ERGQIVKAFVVPR-PGHG------AGELLVR---ELQDFVKRT--
IAPYKYPRAIEFRDSLPRTETGKLQRFRLREGKP---------------------------
